# *HLA* genotype-clinical phenotype correlations in multiple sclerosis and neuromyelitis optica spectrum disorders based on Japan MS/NMOSD Biobank data

**DOI:** 10.1038/s41598-020-79833-7

**Published:** 2021-01-12

**Authors:** Mitsuru Watanabe, Yuri Nakamura, Shinya Sato, Masaaki Niino, Hikoaki Fukaura, Masami Tanaka, Hirofumi Ochi, Takashi Kanda, Yukio Takeshita, Takanori Yokota, Yoichiro Nishida, Makoto Matsui, Shigemi Nagayama, Susumu Kusunoki, Katsuichi Miyamoto, Masanori Mizuno, Izumi Kawachi, Etsuji Saji, Takashi Ohashi, Shun Shimohama, Shin Hisahara, Kazutoshi Nishiyama, Takahiro Iizuka, Yuji Nakatsuji, Tatsusada Okuno, Kazuhide Ochi, Akio Suzumura, Ken Yamamoto, Yuji Kawano, Shoji Tsuji, Makoto Hirata, Ryuichi Sakate, Tomonori Kimura, Yuko Shimizu, Akiko Nagaishi, Kazumasa Okada, Fumie Hayashi, Ayako Sakoda, Katsuhisa Masaki, Koji Shinoda, Noriko Isobe, Takuya Matsushita, Jun-ichi Kira

**Affiliations:** 1 Department of Neurology, Neurological Institute, Graduate School of Medical Sciences, Kyushu University, 3-1-1 Maidashi, Higashi-ku, Fukuoka, 812-8582 Japan; 2 Department of Neurology, Brain and Nerve Center, Fukuoka Central Hospital, 2-6-11 Yakuin, Chuo-ku, Fukuoka, 810-0022 Japan; 3 School of Pharmacy at Fukuoka, International University of Health and Welfare, 137-1 Enokizu, Okawa, Fukuoka 831-8501 Japan; 4 Department of Clinical Research, National Hospital Organization Hokkaido Medical Center, 1-1, 5-7 Yamanote, Nishi-ku, Sapporo, 063-0005 Japan; 5 Department of Neurology, Saitama Medical Center, 1981 Kamoda, Kawagoe, Saitama 350-8550 Japan; 6 Multiple Sclerosis Center, National Hospital Organization Utano National Hospital, 8 Ondoyama, Narutaki, Ukyo-ku, Kyoto, 616-8255 Japan; 7 Kyoto MS Center, Kyoto Min-Iren Chuo Hospital, 2-1 Tsuchimoto-Cho, Kyoto, 616-8147 Japan; 8 Department of Neurology and Geriatric Medicine, Ehime University Graduate School of Medicine, 454 Shitsukawa, Toon, Ehime 791-0295 Japan; 9 Department of Neurology and Clinical Neuroscience, Yamaguchi University Graduate School of Medicine, 1-1-1 Minamikogushi, Ube, Yamaguchi 755-8505 Japan; 10 Department of Neurology and Neurological Science, Tokyo Medical and Dental University, 1-5-45 Yushima, Bunkyo-ku, Tokyo, 113-8519 Japan; 11 Department of Neurology, Kanazawa Medical University, 1-1 Uchinada, Kahoku, Ishikawa 920-0293 Japan; 12 Department of Neurology, Faculty of Medicine, Kindai University, 377-2 Ohno-Higashi, Osaka-Sayama, Osaka 589-8511 Japan; 13 Division of Neurology and Gerontology, Department of Internal Medicine, Iwate Medical University, 2-1-1 Idaidoori, Yahaba, Iwate 028-3695 Japan; 14 Department of Neurology, Brain Research Institute, Niigata University, 1-757 Asahimachi, Chuo-ku, Niigata, 951-8585 Japan; 15 Comprehensive Medical Education Center, Niigata University School of Medicine, 1-757 Asahimachi, Chuo-ku, Niigata, 951-8585 Japan; 16 Division of Neurology, Department of Internal Medicine, Tokyo Women’s Medical University Yachiyo Medical Center, 477-96 Owadashinden, Yachiyo, Chiba 276-8524 Japan; 17 Department of Neurology, Sapporo Medical University School of Medicine, South 1, West 16, Chuo-ku, Sapporo, 060-8543 Japan; 18 Department of Neurology, Kitasato University School of Medicine, 1-15-1 Kitasato, Minami-ku, Sagamihara, Kanagawa 252-0374 Japan; 19 Department of Neurology, Osaka University Graduate School of Medicine, 2-2 Yamadaoka, Suita, Osaka 565-0871 Japan; 20 Department of Neurology, University of Toyama, 2630 Sugitani, Toyama, 930-0194 Japan; 21 Department of Clinical Neuroscience and Therapeutics, Graduate School of Biomedical and Health Sciences, Hiroshima University, 1-2-3 Kasumi, Minami-ku, Hiroshima, 734-8551 Japan; 22 Department of Neurology, Kaikokai-Jousai Hospital, 1-4 Kitabatake, Nakamura-ku, Nagoya, 453-0815 Japan; 23 Department of Medical Biochemistry, Kurume University School of Medicine, 67 Asahi-machi, Kurume, Fukuoka 830-0011 Japan; 24 Department of Neurology, National Hospital Organization Omuta National Hospital, 1044-1 Oaza Tachibana, Omuta, Fukuoka 837-0911 Japan; 25 Department of Neurology, Graduate School of Medicine, The University of Tokyo, 7-3-1 Hongo, Bunkyo-ku, Tokyo, 113-8655 Japan; 26 Laboratory of Rare Disease Resource Library, Center for Rare Disease Research, National Institutes of Biomedical Innovation, Health and Nutrition, 7‑6‑8, Saito‑Asagi, Ibaraki, Osaka 567‑0085 Japan; 27 Department of Neurology, School of Medicine, Tokyo Women’s Medical University, 8-1, Kawada-cho, Shinjuku-ku, Tokyo, 162-8666 Japan; 28 Department of Neurology, National Hospital Organization Nagasaki Kawatana Medical Center, 2005-1 Shimogumigo, Kawatana-machi, Nagasaki, 859-3615 Japan; 29 Department of Neurology, University of Occupational and Environmental Health School of Medicine, 1-1 Iseigaoka, Yahatanishi-ku, Kitakyushu, Fukuoka 807-8555 Japan; 30 Department of Neurological Therapeutics, Neurological Institute, Graduate School of Medical Sciences, Kyushu University, 3-1-1 Maidashi, Higashi-ku, Fukuoka, 812-8582 Japan; 31 Translational Neuroscience Center, Graduate School of Medicine, and School of Pharmacy at Fukuoka, International University of Health and Welfare, 137-1 Enokizu, Okawa, Fukuoka 831-8501 Japan

**Keywords:** Multiple sclerosis, Neuroimmunology, Neurology, Genetic association study

## Abstract

*HLA* genotype-clinical phenotype correlations are not established for multiple sclerosis (MS) and neuromyelitis optica spectrum disorders (NMOSD). We studied *HLA-DRB1/DPB1* genotype–phenotype correlations in 528 MS and 165 NMOSD cases using Japan MS/NMOSD Biobank materials. HLA-DRB1*04:05, DRB1*15:01 and DPB1*03:01 correlated with MS susceptibility and DRB1*01:01, DRB1*09:01, DRB1*13:02 and DPB1*04:01 were protective against MS. HLA-DRB1*15:01 was associated with increased optic neuritis and cerebellar involvement and worsened visual and pyramidal functional scale (FS) scores, resulting in higher progression index values. HLA-DRB1*04:05 was associated with younger onset age, high visual FS scores, and a high tendency to develop optic neuritis. HLA-DPB1*03:01 increased brainstem and cerebellar FS scores. By contrast, HLA-DRB1*01:01 decreased spinal cord involvement and sensory FS scores, HLA-DRB1*09:01 decreased annualized relapse rate, brainstem involvement and bowel and bladder FS scores, and HLA-DRB1*13:02 decreased spinal cord and brainstem involvement. In NMOSD, HLA-DRB1*08:02 and DPB1*05:01 were associated with susceptibility and DRB1*09:01 was protective. Multivariable analysis revealed old onset age, long disease duration, and many relapses as independent disability risks in both MS and NMOSD, and HLA-DRB1*15:01 as an independent risk only in MS. Therefore, both susceptibility and protective alleles can influence the clinical manifestations in MS, while such genotype–phenotype correlations are unclear in NMOSD.

## Introduction

Multiple sclerosis (MS) and neuromyelitis optica spectrum disorders (NMOSD) are inflammatory diseases of the central nervous system (CNS). Although the prevalence of MS is gradually increasing in Japan, it is still low (< 20/100,000) compared with Europe and North America (> 100/100,000)^1–3^. Clinical features are also different between Japanese and Caucasian patients. Disease severity of MS is milder in Japanese compared with Caucasian patients^4,5^. The prevalence of oligoclonal IgG bands (OCBs) was estimated to be lower in Japanese compared with Caucasian MS patients^6,7^. On MRI, cerebellar and parietal lesions were less frequently detected in Japanese compared with European MS patients^5,8^. Moreover, genetic background is also different between these populations. For example, *human leukocyte antigen* (*HLA*)-DRB1*15:01 is the strongest genetic susceptibility factor for MS in Europeans, while both HLA-DRB1*15:01 and DRB1*04:05, which is a rare allele in Northern Europeans, are frequent susceptibility alleles in Japanese MS patients^9–13^. In NMOSD, although the prevalence between Japan and other countries is similar^14^, the genetic risk factors are thought to be different^15^. These observations prompted us to establish a biobank for Japanese MS and NMOSD (Japan MS/NMOSD Biobank) to collect clinical information and biological samples, such as DNA, as a part of the Rare Disease Bank at the National Institutes of Biomedical Innovation, Health and Nutrition in Japan^16^.

Various susceptibility and protective *HLA* class II alleles have been reported in MS and NMOSD, including in Japanese patients^11,13,15,17^. *HLA* genotype-clinical phenotype correlations have been reported, focusing on the major susceptibility alleles in Caucasian (HLA-DRB1*15:01) and Japanese MS patients (HLA-DRB1*15:01 and DRB1*04:05). However, phenotype correlations are unknown in NMOSD. In Caucasian patients with MS, HLA-DRB1*15:01 is associated with younger age at onset, higher white matter lesion volume, greater brain atrophy and impairment of cognitive function^10,18^, but its association with clinical course, disease severity, and prognosis is controversial^19–23^. In Japanese patients with MS, HLA-DRB1*15:01 is associated with a higher frequency of cerebrospinal fluid (CSF) IgG abnormality, including OCBs, while DRB1*04:05 is associated with younger age at onset, lower frequency of CSF IgG abnormality, milder disease course and fewer intracortical lesions^7,11,13,24^. In the present study, we aimed to identify relationships between various susceptibility/protective *HLA* alleles and clinical manifestations in MS and NMOSD using the newly established Japan MS/NMOSD Biobank data.

## Results

### Comparison of demographic features between MS and NMOSD

We enrolled 739 patients and collected 731 DNA, 528 plasma and 566 serum samples. Overall, 19, 8, and 1 patients were excluded from the analyses because of a lack of clinical information, a lack of DNA samples, and double registration, respectively. Clinical information and genotypes of *HLA-DRB1* and *-DPB1* alleles were available from 528 MS and 183 NMO/NMOSD patients based on the 2010 revised McDonald criteria^25^ and NMO and NMOSD criteria advocated by Wingerchuk in 2006 and 2007^26,27^, respectively. Of 183 NMO/NMOSD patients enrolled, 165 patients fulfilled the international consensus diagnostic criteria of NMOSD published in 2015^28^. In the context of the current clinical situation, we conducted the following analyses of NMOSD using data from these 165 patients. MS patients included 438 relapsing–remitting MS (RRMS), 70 secondary progressive MS (SPMS), and 19 primary progressive MS (PPMS) cases. Comparing clinical and laboratory information between MS and NMOSD (Table 1), MS patients were younger at onset and at registration compared with NMOSD patients (both *p* < 0.001). The female to male ratio in NMOSD was higher than in MS (*p* < 0.001). Disease duration was longer in MS than in NMOSD patients (*p* = 0.004). There was no significant difference in the total number of relapses between the two groups, but annualized relapse rate (ARR) was higher in NMOSD than in MS (*p* = 0.001). NMOSD patients experienced optic neuritis more frequently than MS patients, while MS patients had higher frequencies of cerebrum, cerebellum, and brainstem involvement than NMOSD patients. Myelitis, especially episodes of transverse myelitis and the presence of longitudinally extensive spinal cord lesions (LESCLs), was more frequent in NMOSD compared with MS (*p* = 0.031, *p* < 0.001 and *p* < 0.001, respectively). NMOSD patients had higher Kurtzke’s Expanded Disability Status Scale (EDSS) scores^29^ and progression index (PI) values than MS patients (both *p* < 0.001). Of the seven functional system (FS) scores^29^, visual, pyramidal, sensory, and bowel and bladder FS scores were significantly higher in NMOSD than in MS, while brainstem, cerebellar and cerebral FS scores were significantly higher in MS than in NMOSD. Patients with MS more frequently had CSF IgG abnormalities including OCB-positivity and/or high IgG index (> 0.658)^30^ than those with NMOSD (*p* < 0.001). Anti-aquaporin 4 (AQP4) antibody was positive in 95.1% of NMOSD patients. In this study, four cases with anti-AQP4 antibody were diagnosed as MS. These diagnoses were ultimately made by each doctor-in-charge based on the patients’ clinical and MRI characteristics and clinical courses, and they were treated with interferon-β (IFN-β). Excluding these cases from the MS group did not significantly change our findings. Half of the MS patients and one-quarter of the NMOSD patients showed a benign course. Co-existing autoimmune diseases and/or autoantibodies, especially Sjögren syndrome, anti-nuclear antibody (ANA), and anti-SS-A/SS-B antibodies, were more frequent in NMOSD patients compared with MS patients (all *p* < 0.001), while frequencies of co-existing thyroid disease and/or thyroid-related antibodies did not differ between the two groups. Almost all NMOSD patients in the cohort were treated with oral corticosteroids, and two thirds with immunosuppressant drugs. More than 85% of MS patients were treated with disease-modifying drugs (DMDs). Twenty-seven NMOSD patients were treated with IFN-β. Out of the 23 patients for whom data on IFN-β efficacy was obtained, IFN-β was not effective in 18 (78.3%). The other five patients who showed effective treatment responses to IFN-β were additionally treated with corticosteroids.

**Table 1:** Clinical and laboratory characteristics of Japanese patients with MS and NMOSD.

	MS (n = 528)	NMOSD (n = 165)	*p*
Female	368/528 (69.7)	143/165 (86.7)	** < 0.001**
Age at registration, y	41 [34–49]	53 [44–63]	** < 0.001**
Course (RR/SP/PP)	438/70/19 (83.1/13.3/3.6)	–	–
Age at onset, y	30 [23–38]	45 [33–54]	** < 0.001**
Duration of disease, y	9.1 [4.6–15.1]	7.0 [3.4–12.8]	**0.004**
Number of relapses	2 [1–4]	2 [1–5]	0.257
Annualized relapse rate	0.26 [0.13–0.55]	0.41 [0.21–0.75]	**0.001**
Patients with family history of MS or NMOSD	9/517 (1.7)	5/161 (3.1)	0.339
**Lesion sites**			
Optic nerve (optic neuritis)	191/519 (36.8)	98/164 (59.8)	** < 0.001**
Cerebrum	345/521 (66.2)	39/163 (23.9)	** < 0.001**
Cerebellum	109/517 (21.1)	14/163 (8.6)	** < 0.001**
Brain stem	254/517 (49.1)	63/163 (38.7)	**0.020**
Spinal cord	396/520 (76.2)	138/164 (84.2)	**0.031**
**CSF IgG abnormality**	309/440 (70.2)	55/137 (40.2)	** < 0.001**
Presence of OCBs	262/411 (63.7)	20/118 (17.0)	** < 0.001**
High IgG index (> 0.658)	212/374 (56.7)	46/122 (37.7)	** < 0.001**
Anti-AQP4 Ab positivity	4/454 (0.9)	155/163 (95.1)	** < 0.001**
EDSS score	2.0 [1.0–4.0]	3.5 [2.0–5.5]	** < 0.001**
PI	0.22 [0.07–0.49]	0.42 [0.23–0.94]	** < 0.001**
MSSS	2.65 [0.67–5.78]	–	–
FS score*			
Visual functions	0 [0–1]	0 [0–5]	** < 0.001**
Brainstem functions	0 [0–2]	0 [0–1]	**0.015**
Pyramidal functions	1 [0–3]	2 [0–3]	**0.001**
Cerebellar functions	0 [0–2]	0 [0–1]	** < 0.001**
Sensory functions	0 [0–2]	1 [0–4]	** < 0.001**
Bowel and bladder functions	0 [0–1]	0 [0–2]	**0.010**
Cerebral functions	0 [0–1]	0 [0–0]	**0.050**
**Episodes**			
Bilateral optic neuritis	27/516 (5.2)	29/160 (18.1)	** < 0.001**
Transverse myelitis	41/513 (8.0)	64/163 (39.3)	** < 0.001**
**MRI**			
Huge brain lesion > 3 cm	8/522 (1.5)	7/165 (4.2)	0.060
LESCL	10/523 (1.9)	93/162 (57.4)	** < 0.001**
**Benign course**			
EDSS ≤ 2 and disease duration ≥ 10 y	101/234 (43.2)	9/50 (18.0)	**0.001**
EDSS ≤ 3 and disease duration ≥ 10 y	127/234 (54.3)	13/50 (26.0)	** < 0.001**
AID/autoantibodies	77/494 (15.6)	64/155 (41.3)	** < 0.001**
**AID**	31/491 (6.3)	23/151 (15.2)	**0.001**
Sjögren syndrome	7/491 (1.4)	16/151 (10.6)	** < 0.001**
Thyroid diseases (Basedow, Hashimoto)	15/491 (3.1)	3/151 (2.0)	0.586
**Autoantibodies**	66/484 (13.6)	60/148 (40.5)	** < 0.001**
ANA	26/484 (5.4)	29/148 (19.6)	** < 0.001**
Anti-SS-A/SS-B Ab	14/484 (2.9)	31/148 (21.0)	** < 0.001**
Anti-Tg/TPO/TSHR Ab	26/484 (5.4)	14/148 (9.5)	0.083
**PSL/IS**	69/526 (13.1)	162/165 (98.2)	** < 0.001**
PSL	57/509 (11.2)	157/163 (96.3)	** < 0.001**
IS	24/516 (4.7)	111/164 (67.7)	** < 0.001**
**DMD**	459/528 (86.9)	27/165 (16.4)	** < 0.001**
IFN-β	361/528 (68.4)	27/165 (16.4)	** < 0.001**
Fingolimod	217/525 (41.3)	1/164 (0.6)	** < 0.001**

Bold values indicate significant differences.

Values are median [interquartile ranges] or count (%).

*Ab*, antibody, *AID* autoimmune disease, *ANA*, anti-nuclear antibody, *AQP4* aquaporin 4, *CSF* cerebrospinal fluid, *EDSS*, expanded disability status scale, *FS* functional system, *IFN* interferon, *IS* immunosuppressants, *LESCL* longitudinally extensive spinal cord lesion, *MS* multiple sclerosis, *MSSS* multiple sclerosis severity score, *NMOSD* neuromyelitis optica spectrum disorders, *OCBs* oligoclonal IgG bands, *PI* progression index, *PP* primary progressive, *PSL* prednisolone, *RR*, relapsing–remitting, *SP* secondary progressive, *Tg* thyroglobulin, *TPO* thyroid peroxidase, *TSHR* thyroid stimulating hormone receptor, *y* years.

*In the section of functional system scores, values are median [10–90th percentile].

### Comparison of the demographic and clinical features of MS patients between northern and southern Japan

We previously reported a difference in the clinical characteristics of MS patients between northern and southern Japan^1,13^; therefore, MS patients were stratified by their resident area into two groups (northern and southern Japan) at a latitude of 37° North. Of 528 MS patients, 125 and 371 patients lived in northern and southern Japan, respectively (residence information was missing for 32 patients). As shown in Supplementary Table S1, northern MS patients had lower numbers of relapses (*p* < 0.001), annualized relapse rate (*p* = 0.001), and FS score of cerebellar function (*p* = 0.050), but they experienced cerebrum relapses more frequently than southern MS patients (*p* = 0.005). Although disease disability and severity did not differ between northern and southern MS patients, patients with a benign disease course (EDSS ≤ 3 at 10 or more years of disease duration) were more frequently observed in southern Japan than in northern Japan (*p* = 0.027). Patients with a high IgG index were more frequent in northern Japan than in southern Japan (*p* = 0.039), but the OCB positivity rate was similar between groups. Patients who experienced transverse myelitis and autoantibody positivity, especially for ANA and anti-SS-A and/or -SS-B antibodies, were more frequent in southern Japan than in northern Japan (*p* = 0.007, 0.001, 0.001 and 0.026, respectively). Although a small number of PPMS patients were registered only in southern Japan, the above-mentioned differences were similar if these 19 PPMS patients were excluded (data not shown).

### Frequencies of the *HLA-DRB1* and *-DPB1* alleles

As shown in Tables 2 and 3, the carrier frequencies of the HLA-DRB1*04:05, DRB1*15:01, and DPB1*03:01 alleles were higher in MS patients than in healthy controls (corrected *p* [*p*^*corr*^] < 0.001, *p*^*corr*^ < 0.001 and *p*^*corr*^ = 0.006, respectively). Those of HLA-DRB1*01:01, DRB1*09:01, DRB1*13:02 and DPB1*04:01 were lower in MS patients than in healthy controls (*p*^*corr*^ = 0.001, *p*^*corr*^ < 0.001, *p*^*corr*^ < 0.001 and *p*^*corr*^ = 0.006, respectively). In NMOSD patients, the carrier frequencies of HLA-DRB1*08:02 and DPB1*05:01 were higher and those of HLA-DRB1*09:01 were lower than in healthy controls (*p*^*corr*^ = 0.008, *p*^*corr*^ < 0.001 and *p*^*corr*^ < 0.001, respectively).

**Table 2:** Carrier frequencies of *HLA-DRB1* in patients with MS and NMOSD and in healthy controls.

*DRB1*	MS (n = 528)	NMOSD (n = 165)	HCs (n = 394)	MS vs. HCs			NMOSD vs. HCs		
				OR (95% CI)	*p*^*uncorr*^	*p*^*corr*^	OR (95% CI)	*p*^*uncorr*^	*p*^*corr*^
*01:01*	29 (5.5)	25 (15.2)	53 (13.5)	0.38 (0.23–0.60)	** < 0.001**	**0.001**	1.15 (0.69–1.92)	NS	NS
*03:01*	1 (0.2)	1 (0.6)	0	–	NS	NS	–	NS	NS
*04:01*	9 (1.7)	6 (3.6)	9 (2.3)	0.74 (0.29–1.89)	NS	NS	1.61 (0.57–4.61)	NS	NS
*04:03*	46 (8.7)	5 (3.0)	22 (5.6)	1.61 (0.95–2.73)	NS	NS	0.53 (0.20–1.42)	NS	NS
*04:04*	4 (0.8)	0	2 (0.5)	1.50 (0.27–8.21)	NS	NS	–	NS	NS
*04:05*	214 (40.5)	26 (15.8)	100 (25.4)	2.00 (1.51–2.67)	** < 0.001**	** < 0.001**	0.55 (0.34–0.89)	**0.014**	NS
*04:06*	53 (10.0)	5 (3.0)	26 (6.6)	1.58 (0.97–2.57)	NS	NS	0.44 (0.17–1.17)	NS	NS
*04:07*	7 (1.3)	0	4 (1.0)	1.31 (0.38–4.51)	NS	NS	–	NS	NS
*04:10*	29 (5.5)	6 (3.6)	8 (2.0)	2.80 (1.27–6.20)	**0.010**	NS	1.82 (0.62–5.33)	NS	NS
*07:01*	1 (0.2)	2 (1.2)	2 (0.5)	0.37 (0.03–4.12)	NS	NS	2.40 (0.34–17.22)	NS	NS
*08:01*	0	0	1 (0.3)	–	NS	NS	–	NS	NS
*08:02*	54 (10.2)	30 (18.2)	29 (7.4)	1.43 (0.89–2.30)	NS	NS	2.80 (1.62–4.83)	** < 0.001**	**0.008**
*08:03*	85 (16.1)	22 (13.3)	56 (14.2)	1.16 (0.80–1.67)	NS	NS	0.93 (0.55–1.58)	NS	NS
*08:09*	1 (0.2)	0	0	–	NS	NS	NA	NA	NA
*09:01*	76 (14.4)	15 (9.1)	104 (26.4)	0.47 (0.34–0.65)	** < 0.001**	** < 0.001**	0.28 (0.16–0.50)	** < 0.001**	** < 0.001**
*10:01*	5 (1.0)	3 (1.8)	2 (0.5)	1.87 (0.36–9.71)	NS	NS	3.63 (0.60–21.93)	NS	NS
*11:01*	16 (3.0)	10 (6.1)	19 (4.8)	0.62 (0.31–1.22)	NS	NS	1.27 (0.58–2.80)	NS	NS
*12:01*	25 (4.7)	16 (9.7)	38 (9.6)	0.47 (0.28–0.79)	**0.005**	NS	1.01 (0.54–1.86)	NS	NS
*12:02*	8 (1.5)	13 (7.9)	15 (3.8)	0.39 (0.16–0.93)	**0.033**	NS	2.16 (1.00–4.65)	NS (0.055)	NS
*13:01*	1 (0.2)	0	2 (0.5)	0.37 (0.03–4.12)	NS	NS	–	NS	NS
*13:02*	15 (2.8)	21 (12.7)	46 (11.7)	0.22 (0.12–0.40)	** < 0.001**	** < 0.001**	1.10 (0.64–1.92)	NS	NS
*13:03*	0	1 (0.6)	0	NA	NA	NA	–	NS	NS
*13:07*	1 (0.2)	0	1 (0.3)	0.75 (0.05–11.96)	NS	NS	–	NS	NS
*14:02*	0	0	1 (0.3)	–	NS	NS	–	NS	NS
*14:03*	18 (3.4)	7 (4.2)	14 (3.6)	0.96 (0.47–1.95)	NS	NS	1.20 (0.48–3.04)	NS	NS
*14:05*	26 (4.9)	13 (7.9)	15 (3.8)	1.31 (0.68–2.51)	NS	NS	2.16 (1.00–4.65)	NS (0.055)	NS
*14:06*	8 (1.5)	4 (2.4)	6 (1.5)	0.99 (0.34–2.89)	NS	NS	1.61 (0.45–5.77)	NS	NS
*14:07*	0	0	2 (0.5)	–	NS	NS	–	NS	NS
*14:54*	20 (3.8)	14 (8.5)	28 (7.1)	0.51 (0.29–0.93)	**0.035**	NS	1.21 (0.62–2.37)	NS	NS
*15:01*	179 (33.9)	33 (20.0)	67 (17.0)	2.50 (1.82–3.44)	** < 0.001**	** < 0.001**	1.22 (0.77–1.94)	NS	NS
*15:02*	76 (14.4)	31 (18.8)	83 (21.1)	0.63 (0.45–0.89)	**0.010**	NS	0.87 (0.55–1.37)	NS	NS
*16:02*	8 (1.5)	6 (3.6)	4 (1.0)	1.50 (0.45–5.02)	NS	NS	3.68 (1.02–13.21)	NS (0.072)	NS

Bold values indicate significant differences.

Values are numbers (%).

*CI* confidence interval, *HC* healthy control, *MS* multiple sclerosis, *NA* not applicable, *NS* not significant, *OR* odds ratio, *p*^*corr*^ corrected *p* value, *p*^*uncorr*^ uncorrected *p* value.

**Table 3:** Carrier frequencies of *HLA-DPB1* in patients with MS and NMOSD and in healthy controls.

*DPB1*	MS (n = 528)	NMOSD (n = 165)	HCs (n = 394)	MS vs. HCs			NMOSD vs. HCs		
				OR (95%CI)	*p*^*uncorr*^	*p*^*corr*^	OR (95%CI)	*p*^*uncorr*^	*p*^*corr*^
*01:01*	1 (0.2)	0	0	–	NS	NS	NA	NA	NA
*02:01*	197 (37.3)	57 (34.6)	148 (37.6)	0.99 (0.76–1.30)	NS	NS	0.88 (0.60–1.28)	NS	NS
*02:02*	28 (5.3)	15 (9.1)	21 (5.3)	0.99 (0.56–1.78)	NS	NS	1.78 (0.89–3.54)	NS	NS
*03:01*	72 (13.6)	6 (3.6)	25 (6.4)	2.33 (1.45–3.75)	** < 0.001**	**0.006**	0.56 (0.22–1.38)	NS	NS
*04:01*	18 (3.4)	19 (11.5)	36 (9.1)	0.35 (0.20–0.63)	** < 0.001**	**0.006**	1.29 (0.72–2.33)	NS	NS
*04:02*	86 (16.3)	31 (18.8)	73 (18.5)	0.86 (0.61–1.21)	NS	NS	1.02 (0.64–1.62)	NS	NS
*05:01*	367 (69.5)	135 (81.8)	254 (64.5)	1.26 (0.95–1.66)	NS	NS	2.48 (1.59–3.88)	** < 0.001**	** < 0.001**
*06:01*	5 (1.0)	2 (1.2)	8 (2.0)	0.46 (0.15–1.42)	NS	NS	0.59 (0.12–2.82)	NS	NS
*09:01*	73 (13.8)	27 (16.4)	70 (17.8)	0.74 (0.52–1.06)	NS	NS	0.91 (0.56–1.47)	NS	NS
*13:01*	23 (4.4)	4 (2.4)	17 (4.3)	1.01 (0.53–1.92)	NS	NS	0.55 (0.18–1.66)	NS	NS
*14:01*	23 (4.4)	2 (1.2)	11 (2.8)	1.59 (0.76–3.29)	NS	NS	0.43 (0.09–1.95)	NS	NS
*17:01*	1 (0.2)	1 (0.6)	0	–	NS	NS	–	NS	NS
*19:01*	7 (1.3)	1 (0.6)	4 (1.0)	1.31 (0.38–4.51)	NS	NS	0.59 (0.07–5.36)	NS	NS
*22:01*	0	0	1 (0.3)	–	NS	NS	–	NS	NS
*25:01*	1 (0.2)	0	0	–	NS	NS	NA	NA	NA
*36:01*	3 (0.6)	0	0	–	NS	NS	NA	NA	NA
*38:01*	1 (0.2)	0	0	–	NS	NS	NA	NA	NA
*41:01*	0	2 (1.2)	2 (0.5)	–	NS	NS	2.40 (0.34–17.22)	NS	NS

Bold values indicate significant differences.

Values are numbers (%).

*CI* confidence interval, *HC* healthy control, *MS* multiple sclerosis, *NA* not applicable, *NS* not significant, *OR* odds ratio, *p*^*corr*^ corrected *p* value, *p*^*uncorr*^ uncorrected *p* value.

### Comparison of clinical and laboratory characteristics between risk HLA-allele-positive and -negative patients

The main findings of the *HLA* genotype-clinical phenotype correlations are summarized in Tables 4 and 5.

**Table 4:** Summary of *HLA* genotype-clinical phenotype correlations in MS.

HLA allele	Confer susceptibility or resistance	Clinical and laboratory findings	Effects on clinical phenotype
DRB1*04:05	Susceptibility	Age at onset	Younger
		Optic nerve involvement (optic neuritis)	Increased
		Visual FS	Increased
		CSF IgG abnormalities	Decreased
		MSSS (excluding PPMS in southern Japan)	Decreased
DRB1*15:01	Susceptibility	Optic nerve involvement (optic neuritis)	Increased
		Cerebellum involvement	Increased
		Visual FS	Increased
		Pyramidal FS	Increased
		PI	Increased
		CSF IgG abnormalities	Increased
		Anti-SS-A/SS-B Ab	Increased
DPB1*03:01	Susceptibility	Brainstem FS	Increased
		Cerebellar FS	Increased
DRB1*01:01	Resistance	Spinal cord involvement	Decreased
		Sensory FS	Decreased
DRB1*09:01	Resistance	ARR	Decreased
		Brainstem involvement	Decreased
		Bowel and bladder FS	Decreased
DRB1*13:02	Resistance	Spinal cord involvement	Decreased
		Brainstem involvement	Decreased
		OCBs	Decreased
DPB1*04:01	Resistance	OCBs	Decreased

If the effects were derived from only 5 or less than 5 positive cases, such findings were regarded as too preliminary, and therefore they are not described in the table.

*Ab* antibody, *CSF* cerebrospinal fluid, *FS* functional system, *LESCL* longitudinally extensive spinal cord lesion, *MS* multiple sclerosis, *OCBs* oligoclonal IgG bands, *PI* Progression Index.

**Table 5:** Summary of *HLA* genotype-clinical phenotype correlations in NMOSD.

HLA allele	Confer susceptibility or resistance	Clinical and laboratory findings	Effects on clinical phenotype
DRB1*08:02	Susceptibility	OCBs	Decreased
		AID/autoantibodies	Increased
		Autoantibodies	Increased
		Anti-SS-A/SS-B Ab	Increased
DPB1*05:01	Susceptibility	PI	Decreased (due to long disease duration?)
		AID	Increased
		Sjögren syndrome	Increased
		Anti-SS-A/SS-B Ab	Increased
DRB1*09:01	Resistance	AID	Increased
		Sjögren syndrome	Increased

If the effects were derived from only 5 or less than 5 positive cases, such findings were regarded as too preliminary, and therefore they are not described in the table.

*Ab* antibody, *AID* autoimmune disease, *ANA* anti-nuclear antibody, *NMOSD* neuromyelitis optica spectrum disorders, *OCBs* oligoclonal IgG bands, *PI* Progression Index.

#### MS patients

Concerning the susceptibility alleles, MS patients with HLA-DRB1*04:05 were younger at disease onset (*p* = 0.017), had lower frequencies of CSF IgG abnormalities, including presence of OCBs and high IgG index (all *p* < 0.001), and higher visual FS scores (*p* = 0.001), and tended to have optic nerve involvement (optic neuritis) more frequently than patients without DRB1*04:05 (*p* = 0.052) (Table 6). Because disease duration differed between the two groups, the frequency of patients who experienced relapses with optic neuritis and FS scores (≥ 1) of visual function were compared, adjusting for disease duration. The frequencies of patients who experienced optic neuritis tended to be higher in patients with HLA-DRB1*04:05 than those without the allele, but this did not reach statistical significance (odds ratio [OR] 1.33 [95% confidence interval [CI] 0.92–1.94], *p* = 0.132). Regarding visual function, HLA-DRB1*04:05 was an independent risk factor for functional disability of the optic nerve even after adjusting for disease duration (OR 2.30 [95% CI 1.29–4.10], *p* = 0.005).

**Table 6:** Comparison of the clinical characteristics between MS patients with and without HLA-DRB1*04:05.

	HLA-DRB1*04:05		*p*
	Positive (n = 214)	Negative (n = 314)	
Female	146/214 (68.2)	222/314 (70.7)	0.564
Age at registration, y	41 [33–52]	41 [35–48]	0.916
Course (RR/SP/PP)	174/30/10 (81.3/14.1/4.7)	264/40/9 (84.3/12.8/2.9)	0.496
Age at onset, y	28 [22–38]	31 [24–39]	**0.017**
Duration of disease, y	10.3 [5.7–16.8]	8.3 [4.1–14.0]	**0.006**
Number of relapses	2 [1–4]	2 [1–4]	0.159
Annualized relapse rate	0.26 [0.11–0.52]	0.27 [0.13–0.57]	0.370
**Lesion sites**			
Optic nerve (optic neuritis)	89/212 (42.0)	102/307 (33.2)	0.052
Cerebrum	142/212 (67.0)	203/309 (65.7)	0.778
Cerebellum	48/210 (22.9)	61/307 (19.9)	0.443
Brainstem	111/210 (52.9)	143/307 (46.6)	0.179
Spinal cord	163/211 (77.3)	233/309 (75.4)	0.676
Patients with family history of MS or NMOSD	5/211 (2.4)	4/306 (1.3)	0.497
**CSF IgG abnormality**	103/179 (57.5)	206/261 (78.9)	** < .001**
Presence of OCBs	80/166 (48.2)	182/245 (74.3)	** < .001**
High IgG index (> 0.658)	64/151 (42.4)	148/223 (66.4)	** < .001**
Anti-AQP4 Ab positivity	1/182 (0.5)	3/272 (1.1)	0.653
EDSS score	2.0 [1.0–4.5]	2.0 [1.0–3.5]	0.615
PI	0.19 [0.06–0.45]	0.24 [0.08–0.49]	0.136
MSSS	2.44 [0.65–5.84]	2.84 [0.82–5.76]	0.449
FS score*			
Visual functions	0 [0–2]	0 [0–0]	**0.001**
Brainstem functions	0 [0–2]	0 [0–2]	0.375
Pyramidal functions	1 [0–3]	1 [0–3]	0.733
Cerebellar functions	0 [0–3]	0 [0–2]	0.156
Sensory functions	0 [0–3]	0 [0–2]	0.827
Bowel and bladder functions	0 [0–2]	0 [0–1]	0.820
Cerebral functions	0 [0–1]	0 [0–1]	0.855
**Episodes of**			
Bilateral optic neuritis	15/208 (7.2)	12/308 (3.9)	0.109
Transverse myelitis	16/206 (7.8)	25/307 (8.1)	1
**MRI**			
Huge brain lesion > 3 cm	5/212 (2.4)	3/310 (1.0)	0.280
LESCL	3/214 (1.4)	7/309 (2.3)	0.538
**Benign course**			
EDSS ≤ 2 and disease duration ≥ 10 y	49/111 (44.1)	52/123 (42.3)	0.793
EDSS ≤ 3 and disease duration ≥ 10 y	58/111 (52.3)	69/123 (56.1)	0.600
AID/autoantibodies	35/200 (17.5)	42/294 (14.3)	0.377
**AID**	12/198 (6.1)	19/293 (6.5)	1
Sjögren syndrome	1/198 (0.5)	6/293 (2.0)	0.250
Thyroid diseases (Basedow, Hashimoto)	6/198 (3.0)	9/293 (3.1)	1
**Autoantibodies**	31/196 (15.8)	35/288 (12.2)	0.281
ANA	15/196 (7.7)	11/288 (3.8)	0.099
Anti-SS-A/SS-B Ab	4/196 (2.0)	10/288 (3.5)	0.419
Anti-Tg/TPO/TSHR Ab	13/196 (6.6)	13/288 (4.5)	0.313
**PSL/IS**	28/212 (13.2)	41/314 (13.1)	1
PSL	22/205 (10.7)	35/304 (11.5)	0.886
IS	11/209 (5.3)	13/307 (4.2)	0.672
**DMD**	185/214 (86.4)	274/314 (87.3)	0.794
IFN-β	144/214 (67.3)	217/314 (69.1)	0.703
fingolimod	90/212 (42.5)	127/313 (40.6)	0.718

Bold values indicate significant differences.

Values are median [interquartile ranges] or count (%).

*Ab*, antibody, *AID* autoimmune disease, *ANA*, anti-nuclear antibody, *AQP4* aquaporin 4, *CSF* cerebrospinal fluid, *EDSS*, expanded disability status scale, *FS* functional system, *IFN* interferon, *IS* immunosuppressants, *LESCL* longitudinally extensive spinal cord lesion, *MS* multiple sclerosis, *MSSS* multiple sclerosis severity score, *NMOSD* neuromyelitis optica spectrum disorders, *OCBs* oligoclonal IgG bands, *PI* Progression Index, *PP* primary progressive, *PSL* prednisolone, *RR*, relapsing–remitting, *SP* secondary progressive, *Tg* thyroglobulin, *TPO* thyroid peroxidase, *TSHR* thyroid stimulating hormone receptor, *y* years.

*In the section of functional system scores, values are median [10-90th percentile].

We previously reported that the effect of HLA-DRB1*04:05 on MS patients differed between northern and southern Japan^13^; therefore, we additionally compared MS patients from northern and southern Japan with and without HLA-DRB1*04:05 (Supplementary Tables S2, S3). CSF IgG abnormality was less frequent in HLA-DRB1*04:05-positive than -negative patients from northern or southern Japan (*p* = 0.005 and *p* < 0.001, respectively) similar to that for all of Japan. In southern Japan, MS patients with HLA-DRB1*04:05 were younger at disease onset (*p* = 0.009) with higher visual FS scores (*p* = 0.003) and tended to have optic nerve involvement (optic neuritis) more frequently than patients without DRB1*04:05 (*p* = 0.090). In northern Japan, HLA-DRB1*04:05-positive patients had higher pyramidal FS scores (*p* = 0.029) and experienced fingolimod treatment more frequently than HLA-DRB1*04:05-negative patients (*p* = 0.006). Moreover, when excluding PPMS, as in a previous study^13^, PI and MS severity scores (MSSS)^31^ were lower in southern MS patients with HLA-DRB1*04:05 than in those without the allele (*p* = 0.22 and 0.050, respectively) (Supplementary Table S4). When we compared the clinical characteristics of MS patients with HLA-DRB1*04:05 (excluding PPMS) between northern and southern Japan, northern patients had a lower number of relapses (*p* < 0.001) and annualized relapse rate (*p* = 0.006) but higher PI (*p* = 0.018) and MSSS (*p* = 0.008). Patients who experienced transverse myelitis and who had ANA were more frequent in southern Japan than in northern Japan (*p* = 0.023 and 0.012, respectively). Southern MS patients with HLA-DRB1*04:05 were less frequently treated with DMD, especially with fingolimod, compared with northern patients (*p* = 0.018 and 0.009, respectively) (Supplementary Table S5).

HLA-DRB1*15:01-positive MS patients had CSF IgG abnormalities more commonly than DRB1*15:01-negative patients (*p* = 0.036) (Table 7) and they experienced attacks in the optic nerve (optic neuritis) and cerebellum more frequently (*p* = 0.044 and 0.031, respectively), and also tended to have a higher FS score of visual function (*p* = 0.060) compared with those without the allele. FS scores of cerebellar function were similar between these two groups. FS scores of pyramidal function and PI were significantly higher in DRB1*15:01-positive patients than in DRB1*15:01-negative patients (*p* = 0.027 and 0.031, respectively), although EDSS scores and MSSS were not significantly different between them. MS patients with DRB1*15:01 had anti-SS-A and/or -SS-B antibodies more frequently than those without (*p* = 0.007). HLA-DPB1*03:01 carriers had higher FS scores of brainstem and cerebellar function than non-carriers (*p* = 0.035 and 0.004, respectively) (Supplementary Table S6).

**Table 7:** Comparison of the clinical characteristics between MS patients with and without HLA-DRB1*15:01.

	HLA-DRB1*15:01		*p*
	Positive (n = 179)	Negative (n = 349)	
Female	128/179 (71.5)	240/349 (68.8)	0.549
Age at registration, y	40 35–47]	42 [34–51]	0.362
Course (RR/SP/PP)	149/22/8 (83.2/12.3/4.5)	289/48/11 (83.0/13.8/3.2)	0.683
Age at onset, y	30 [23–37]	30 [23–40]	0.776
Duration of disease, y	9.0 [4.0–14.7]	9.1 [5.4–15.1]	0.398
Number of relapses	2 [1–4]	2 [1–4]	0.908
Annualized relapse rate	0.30 [0.15–0.62]	0.25 [0.12–0.54]	0.217
**Lesion sites**			
Optic nerve (optic neuritis)	76/177 (42.9)	115/342 (33.6)	**0.044**
Cerebrum	122/177 (68.9)	223/344 (64.8)	0.379
Cerebellum	47/177 (26.6)	62/340 (18.2)	**0.031**
Brainstem	90/177 (50.8)	164/340 (48.2)	0.580
Spinal cord	137/177 (77.4)	259/343 (75.5)	0.665
Patients with family history of MS or NMOSD	3/174 (1.7)	6/343 (1.7)	1
**CSF IgG abnormality**	112/146 (76.7)	197/294 (67.0)	**0.036**
Presence of OCBs	104/139 (74.8)	158/272 (58.1)	**0.001**
High IgG index (> 0.658)	80/122 (65.6)	132/252 (52.4)	**0.019**
Anti-AQP4 Ab positivity	4/146 (2.7)	0/308 (0)	**0.010**
EDSS score	2.0 [1.0–4.5]	2.0 [1.0–4.0]	0.284
PI	0.28 [0.09–0.53]	0.20 [0.05–0.44]	**0.031**
MSSS	2.93 [0.85–5.97]	2.34 [0.65–5.58]	0.082
FS score*			
Visual functions	0 [0–1]	0 [0–0]	0.060
Brainstem functions	0 [0–2]	0 [0–2]	0.362
Pyramidal functions	1 [0–3]	1 [0–3]	**0.027**
Cerebellar functions	0 [0–2]	0 [0–2]	0.139
Sensory functions	1 [0–3]	0 [0–2]	0.204
Bowel and bladder functions	0 [0–2]	0 [0–1]	0.499
Cerebral functions	0 [0–1]	0 [0–1]	0.867
**Episodes of**			
Bilateral optic neuritis	11/176 (6.3)	16/340 (4.7)	0.532
Transverse myelitis	14/173 (8.1)	27/340 (7.9)	1
**MRI**			
Huge brain lesion > 3 cm	0/175 (0)	8/347 (2.3)	0.057
LESCL	3/175 (1.7)	7/348 (2.0)	1
**Benign course**			
EDSS ≤ 2 and disease duration ≥ 10 y	34/79 (43.0)	67/155 (43.2)	1
EDSS ≤ 3 and disease duration ≥ 10 y	44/79 (55.7)	83/155 (53.5)	0.783
AID/autoantibodies	26/168 (15.5)	51/326 (15.6)	1
**AID**	11/168 (6.5)	20/323 (6.2)	0.848
Sjögren syndrome	4/168 (2.4)	3/323 (0.9)	0.238
Thyroid diseases (Basedow, Hashimoto)	7/168 (4.2)	8/323 (2.5)	0.407
**Autoantibodies**	24/166 (14.5)	42/318 (13.2)	0.780
ANA	9/166 (5.4)	17/318 (5.3)	1
Anti-SS-A/SS-B Ab	10/166 (6.0)	4/318 (1.3)	**0.007**
Anti-Tg/TPO/TSHR Ab	8/166 (4.8)	18/318 (5.7)	0.833
**PSL/IS**	20/179 (11.2)	49/347 (14.1)	0.414
PSL	16/174 (9.2)	41/335 (12.2)	0.374
IS	7/172 (4.1)	17/344 (4.9)	0.825
**DMD**	155/179 (86.6)	304/349 (87.1)	0.892
IFN-β	118/179 (65.9)	243/349 (69.6)	0.429
fingolimod	74/179 (41.3)	143/346 (41.3)	1

Bold values indicate significant differences.

Values are median [interquartile ranges] or count (%).

*Ab*, antibody, *AID* autoimmune disease, *ANA*, anti-nuclear antibody, *AQP4* aquaporin 4, *CSF* cerebrospinal fluid, *EDSS*, expanded disability status scale, *FS* functional system, *IFN* interferon, *IS* immunosuppressants, *LESCL* longitudinally extensive spinal cord lesion, *MS* multiple sclerosis, *MSSS* multiple sclerosis severity score, *NMOSD* neuromyelitis optica spectrum disorders, *OCBs* oligoclonal IgG bands, *PI* Progression Index, *PP* primary progressive, *PSL* prednisolone, *RR*, relapsing–remitting, *SP* secondary progressive, *Tg* thyroglobulin, *TPO* thyroid peroxidase, *TSHR* thyroid stimulating hormone receptor, *y* years.

*In the section of functional system scores, values are median [10-90th percentile].

With respect to resistance alleles, MS patients with the HLA-DRB1*01:01 allele had a lower female to male ratio than those without (*p* = 0.006) (Supplementary Table S7). HLA-DRB1*01:01 carriers had spinal cord involvement less frequently and had a lower FS score of sensory function than non-carriers (*p* = 0.022 and 0.048, respectively). HLA-DRB1*09:01-positive MS patients had lower ARR and FS scores of bowel and bladder function and had brainstem involvement less frequently than DRB1*09:01-negative patients (*p* = 0.047, 0.015 and 0.005, respectively) (Supplementary Table S8). EDSS scores, PI and MSSS were not different between these two groups. MS patients with HLA-DRB1*13:02 tended to have brainstem and spinal cord involvement less frequently than those without the allele (*p* = 0.055 and 0.058, respectively). Moreover, HLA-DRB1*13:02-carriers had a lower frequency of OCB-positivity than non-carriers (*p* = 0.002) (Supplementary Table S9). MS patients with the HLA-DPB1*04:01 allele had OCBs less frequently and higher frequencies of LESCL compared with those without the allele (*p* = 0.004 and 0.043, respectively) (Supplementary Table S10).

#### NMOSD patients

With respect to susceptibility alleles, NMOSD patients with the HLA-DRB1*08:02 allele had lower frequencies of OCB-positivity compared with those without the allele (*p* = 0.012) (Supplementary Table S11). Moreover, HLA-DRB1*08:02 carriers were more prone to having autoimmune diseases and/or autoantibodies, especially anti-SS-A and/or -SS-B antibodies, compared with non-carriers (*p* = 0.033 and 0.035, respectively). NMOSD patients with HLA-DPB1*05:01 had longer disease durations and a larger number of relapses compared with those without (*p* = 0.016 and 0.001, respectively) (Supplementary Table S12), while the ARR was comparable between them. Moreover, they tended to have more frequent involvement of the cerebrum and cerebellum compared with those without (*p* = 0.090 and 0.077, respectively). PI was lower in patients with the HLA-DPB1*05:01 allele compared with those without (*p* = 0.034), although EDSS scores were not statistically different. They had Sjögren syndrome and anti-SS-A and/or -SS-B antibodies more often than DPB1*05:01-negative patients (*p* = 0.044 and 0.028, respectively). With respect to protective alleles, HLA-DRB1*09:01-positive NMOSD patients had co-existing autoimmune diseases, especially Sjögren syndrome, more frequently than DRB1*09:01-netagive patients (*p* = 0.030 and 0.034, respectively) (Supplementary Table S13).

### Factors associated with disability

Finally, we evaluated which clinical parameters, laboratory findings and *HLA* subtypes were associated with disability. In RRMS, we focused on HLA-DRB1*04:05 and DRB1*15:01 as genetic factors. Multivariable models showed that old age at onset, long disease duration, many relapses, and being a carrier of the HLA-DRB1*15:01 allele were independent risk factors for high EDSS scores (Table 8). For NMOSD, old age at onset, long disease duration and many relapses were risk factors for high EDSS scores, but episodes of bilateral optic neuritis, experience of LESCL and harbouring the HLA-DPB1*05:01 allele were not (Supplementary Table S14).

**Table 8:** Clinical and genetic factors associated with EDSS scores in RRMS.

EDSS score (ordinal scale)	Univariable		Multivariable	
	Std β	*p*	Std β	*p*
Sex [female/male]	0.004	0.931	− 0.051	0.296
Age at onset (y)	0.090	0.060	0.218	** < 0.001**
Disease duration (y)	0.245	** < 0.001**	0.137	**0.016**
Number of relapses	0.307	** < 0.001**	0.281	** < 0.001**
CSF IgG abnormality [+/−]	0.052	0.321	0.076	0.135
any DMD [treated/non-treated]	0.061	0.202	0.069	0.165
HLA-DRB1*04:05 [+/−]	− 0.005	0.916	0.028	0.589
HLA-DRB1*15:01 [+/−]	0.076	0.111	0.106	**0.037**

Bold values indicate significant correlations.

*CSF* cerebrospinal fluid, *DMD* disease modifying drug, *EDSS* expanded disability status scale, *RRMS* relapsing–remitting multiple sclerosis, *Std* standardized, *y* years.

## Discussion

We used the Japan MS/NMOSD Biobank and the largest cohort of Japanese MS and NMOSD patients studied to date to show that HLA-DRB1*04:05, DRB1*15:01 and DPB1*03:01 are susceptibility alleles, while HLA-DRB1*01:01, DRB1*09:01, DRB1*13:02 and DPB1*04:01 are protective alleles for MS, which is consistent with previous Japanese studies on MS^11,13^. For NMOSD, HLA-DRB1*08:02 and DPB1*05:01 are susceptibility alleles and DRB1*09:01 is a protective allele. This is a novel finding for HLA-DRB1*08:02 and confirmatory for DPB1*05:01 and DRB1*09:01^15^. Importantly, we discovered new significant *HLA* genotype-clinical phenotype correlations in MS and NMOSD.

In our MS cohort, the susceptibility alleles, DRB1*15:01, and DPB1*03:01, were associated overall with worsening functional disability scores. The finding that HLA-DRB1*15:01 increased the involvement of the optic nerve (optic neuritis) and cerebellum and worsened visual and pyramidal FS scores, resulting in greater PI, is a new finding in Asians and is compatible with several reports in Caucasians showing that DRB1*15:01 is associated with greater MSSS and more severe cognitive impairment^18,20^. The relationship of HLA-DRB1*04:05 to clinical manifestations has not been reported in Caucasians, primarily because this allele is rare in most European descendants^32,33^. MSSS was lower in southern MS patients with HLA-DRB1*04:05 than in those without the allele when excluding PPMS in this study, which is in accord with a previous finding that HLA-DRB1*04:05 was associated with a milder disease course as evaluated by EDSS scores and PI in Japanese MS patients, particularly from southern Japan^11,13^. Unexpectedly, we also found an association of this allele with increased tendency of involvement of the optic nerve (optic neuritis) and worsening of visual FS scores. Although these results appear contradictory, the fact that motor disability involving pyramidal and cerebellar functions is more contributory to EDSS scores than other systems, such as visual function, may explain this discrepancy. A comparison of the clinical characteristics of MS patients with HLA-DRB1*04:05 between northern and southern Japan showed that southern patients had a milder disease course and atypical presentation including episodes of transverse myelitis and having ANA, which may result in the lower frequency of DMD treatment. Given that they share HLA-DRB1*04:05 as a common genetic background, environmental factors including residential latitude may contribute in part to these differences in the clinical manifestations. In Caucasians, HLA-DRB1*15:01 has repeatedly been shown to be associated with younger age at onset in MS patients^10,18,34^, while in Japanese, HLA-DRB1*04:05 is associated with a younger age at onset^11,13^, which was confirmed in the present study. Thus, the contribution of *HLA* alleles to a younger age at onset could vary according to race. The association of HLA-DPB1*03:01 with an increase in brainstem and cerebellar FS scores is a novel finding, which further supports a contribution of the susceptibility alleles to aggravation of disease severity in Japanese MS patients.

In contrast, the resistance alleles, HLA-DRB1*01:01, DRB1*09:01 and DRB1*13:02, were associated with decreased functional disability in the present cohort. HLA-DRB1*01:01 decreased involvement of the spinal cord and sensory FS scores, DRB1*09:01 decreased ARR, involvement of the brainstem and bowel and bladder FS scores, and *DRB1:13:02* decreased involvement of the spinal cord and brainstem. HLA-DRB1*01 was reported to decrease PI and the frequency of malignant subtype and to extend the time to reach an EDSS score of 6 in Caucasian patients^35^, which is in line with our findings. Another resistance allele, HLA-DPB1*04:01, was reported to be associated with a reduced MSSS under corticosteroid or azathioprine in Brazilian patients^36^. This was not obvious in the present study, possibly because of the low frequency of HLA-DPB1*04:01 in this cohort. Collectively, these resistance alleles are indicated to decrease disease susceptibility and to decrease involvement of relevant CNS sites in different races.

We examined CSF IgG in our cohort and found that the resistance alleles, HLA-DRB1*13:02 and DPB1*04:01, decreased OCBs while one of the susceptibility alleles, DRB1*15:01, increased, and another, DRB1*04:05, decreased CSF IgG abnormalities. Potentiation of CSF IgG abnormalities by DRB1*15:01 was reported in both Caucasians^9,37,38^ and Japanese patients with MS^11,13,39^, while mitigation of CSF IgG abnormalities by DRB1*04:05 has been repeatedly reported in Japanese patients with MS^7,11,13,39^. Collectively, even for susceptibility alleles, their influence on CSF IgG abnormalities, including OCBs, appears to be heterogeneous. The association of two resistance alleles, HLA-DRB1*13:02 and DPB1*04:01, with lower OCB frequency is a novel finding, which should be confirmed by a large scale study. HLA-DRB1*13:02 is a protective allele for systemic and organ-specific autoimmune diseases, such as rheumatoid arthritis, systemic lupus erythematosus, psoriasis, and autoimmune hepatitis^40^. Therefore, a person with HLA-DRB1*13:02 may have resistance to producing autoreactive immunoglobulins. Only a few studies of Caucasians reported such associations of distinct *HLA* alleles with CSF IgG abnormalities, which may be partly accounted for by the extremely high overall prevalence of OCBs (> 90%)^9,41^ that obscures the effects of *HLA* alleles. Thus, investigation of a Japanese MS cohort, in which the positivity rate of OCBs is not very high (around 60%)^6,7^, for any association of *HLA* alleles with CSF IgG abnormalities is warranted.

Multivariable analysis also revealed HLA-DRB1*15:01 to be an independent risk factor for high EDSS scores in addition to old age at onset, long disease duration, and many relapses. Older age at onset, male sex, and high relapse rate especially in the early disease course, are well known poor prognostic factors in Caucasians^42–45^, but the natural history of MS in Asia is less well studied. We previously assessed the association between age at onset and disease severity using MSSS^13^. Old age at onset is thought to be an independent risk factor for increased disease severity and disability in both Caucasians and Japanese. In contrast to previous studies of Caucasian MS patients^42–44^, this and our previous studies did not show an effect of sex on disease severity or disability in Japanese MS patients^13^. Therefore, male sex can be a poor prognostic factor for Caucasians but not Japanese, although this should be confirmed in a large study, such as a nation-wide survey. The effect of the total number of relapses on prognosis is controversial^46^, but relapse frequency up to year 5 was reported to be predictive in some studies^44,47^. The median disease duration in RRMS patients was 8 years in our study, which may reflect relatively early disease activity.

In NMOSD, no association between any *HLA* allele and clinical manifestations has been found. The present NMOSD patients had highly comparable demographic and clinical features, including sex ratio, age of onset, and frequency of OCB-positivity with previous Japanese clinical studies^48,49^. One of the susceptibility alleles, HLA-DPB1*05:01, was associated with lower PI compared with HLA-DPB1*05:01-negative patients, even though EDSS scores did not differ between them. Because the disability of NMOSD patients is mainly caused by acute attacks, and a progressive disease course is uncommon in NMOSD compared with MS^50–52^, the PI decreases as the disease duration increases, particularly when treatments such as corticosteroids reduce relapses^53^. Indeed, NMOSD patients with DPB1*05:01 had a significantly longer disease duration than those without the allele in our cohort, which confirms this idea. Multivariable analyses also supported the idea that the HLA-DPB1*05:01 allele has no significant impact on disability.

We found that the susceptibility alleles, HLA-DRB1*08:02 and DPB1*05:01, increased the frequencies of concomitant autoimmune diseases and autoantibodies such as Sjögren syndrome and anti-SS-A/SS-B antibodies, while the resistance allele, DRB1*09:01, was also associated with increased frequencies of co-existing autoimmune diseases, such as Sjögren syndrome. Thus, how associations of either the susceptibility or protective alleles with autoimmune traits modulate NMOSD susceptibility or resistance through autoimmune backgrounds remain elusive.

In our multivariable analyses, while higher age at onset, longer disease duration, and more relapses were independent risks for disability, the susceptibility allele, HLA-DPB1*05:01, was not an independent risk factor for disability, in contrast to MS. HLA-DPB1*05:01 could increase the susceptibility to NMOSD but may not confer disability. Associations between older age at onset and higher EDSS scores and between death and high ARR have been reported in European and Asian cohorts^52,54–56^. Collectively, older age at onset and relapse number are regarded as risk factors for disability, underscoring the importance of early introduction of efficacious preventative therapies in elderly-onset NMOSD patients. Although chronic progression is not considered to contribute to NMOSD, the longer disease duration was also an independent risk for disability in our NMOSD patients. We have reported a persistent increase of serum neurofilament light chain in the remission phase in a fraction of NMOSD patients, indicating continuous damage to axons following acute relapses^57^. Long term follow-up studies are required to elucidate whether, and if so how, a longer disease duration influences disability in NMOSD.

In terms of disability in NMOSD patients, our cohort showed relatively mild disability compared with the clinical course of NMOSD patients described by Wingerchuk et al.^27^. However, NMOSD patients without LESCL were reported to have a milder disease course than those with LESCL^58^. Because patients without LESCL occupied 57.4% of NMOSD patients in our cohort, overall disability in our NMOSD cases appeared to be mild. Moreover, a recent study showed that Japanese NMOSD patients with AQP4-IgG had a lower risk of relapse, especially transverse myelitis and optic neuritis attacks, compared with Caucasian patients^52^. This might explain why our cohort had lower EDSS scores and lower frequencies of LESCL than expected values based on the results of Caucasian NMOSD patients. In addition, more than 95% of NMOSD patients received prednisolone treatment, which can decrease disease severity by preventing relapses^53^. Therefore, we think that our cohort showed normal attributes for Japanese NMOSD patients.

In this study, we compared the demographic and clinical features of MS and NMOSD patients and assessed their distinct genetic backgrounds and the differential effects of *HLA* class II alleles on clinical features between these two diseases. Although MS and NMOSD are distinct diseases, both are inflammatory diseases affecting CNS tissues and their clinical findings sometimes overlap. Moreover, it is occasionally difficult to distinguish between these two diseases, particularly for cases of seronegative NMOSD in clinical settings. Thus, we consider that the collection of MS and NMOSD information and biosamples by the Japan MS/NMOSD biobank will be very useful for the characterization of clinical and laboratory findings to differentiate between the two conditions. By describing the differences in clinical and laboratory tests between MS and NMOSD concurrently, the differential aspects of these two diseases will become clearer.

There are several limitations in our study. First, susceptibility *HLA* alleles for MS and NMOSD, other than *HLA-DRB1* and -*DPB1*, were recently reported for Japanese patients using next-generation sequencing^17^. These include HLA-B*39:01 and HLA-B*15:01 for MS and HLA-DQA1*05:03 for NMOSD^17^. We genotyped only two classical *HLA* class II genes (*DRB1* and *DPB1*) using sequence-specific oligonucleotide hybridization. By applying new methodology, such as next-generation sequencing, to this newly established Japan MS/NMOSD Biobank of samples, new risk genes of MS or NMOSD could be identified in the future. Second, healthy controls were not included in the MS/NMOSD Biobank; therefore, we used data of healthy controls collected by the Japan Multiple Sclerosis Genetics Consortium^13^. Therefore, similar risk and resistance alleles to those in the previous study may tend to be obtained; however, the *HLA* genotype-clinical phenotype correlations were newly discovered in the present study. Third, in this MS/NMOSD biobank, the number of enrolled MS patients from southern Japan were approximately 3 times higher than that from northern Japan despite the higher prevalence rate of MS in northern Japan^59^. However, the general population of southern Japan is about 7 times higher than that of northern Japan. Therefore, although potential enrolment bias cannot be completely excluded, we do not think that the 3:1 ratio of enrolled MS patients between southern and northern Japan distorted the results of the present study. Fourth, as we did not systematically examine anti-myelin oligodendrocyte glycoprotein (MOG) antibodies, some patients with anti-MOG antibodies may be included in our MS cohort. However, most adult patients with typical MS, including Japanese, are negative for anti-MOG antibodies^60,61^; therefore, possible contamination of MOG antibody disease would not severely distort our results.

In conclusion, based on the Japan MS/NMOSD Biobank data, we show *HLA* genotype-clinical phenotype correlations concerning the confirmed and newly identified susceptibility and resistance *DRB1* and *DPB1* genes in Japanese patients with MS or NMOSD. The susceptibility alleles were mostly associated with worsening of disease severity, whereas the resistance alleles were associated with amelioration of the disease in MS. By contrast, such genotype–phenotype correlations were not obvious in NMOSD. Together these findings indicate that both susceptibility and resistance alleles can influence the clinical manifestations in MS but not in NMOSD.

## Patients and methods

### Participants

The MS/NMOSD Biobank of Japan was established in 2013 as a part of the Rare Disease Bank at the National Institute of Biomedical Innovation, Health and Nutrition in Japan^16^. Japanese patients with MS and NMOSD, consenting to be registered in this biobank, were recruited from 20 institutes in Japan. MS was diagnosed using the 2010 revised McDonald criteria^25^. The diagnoses of NMO and NMOSD were based on the criteria advocated by Wingerchuk in 2006 and 2007^26,27^. Diagnoses were recorded at the time informed consent was provided by the participants. However, given that the NMOSD criteria updated in 2015 are now widely used and that the items of NMOSD proposed in 2007 are relatively complicated, we limited our analyses to the 165 NMOSD patients who fulfilled the criteria of Wingerchuk 2015^28^ based on the data collected. Participants’ demographic data, clinical information and biological samples (sera, plasma and DNA) were also collected. The biological samples were taken between December 2002 and March 2017. Among 739 patients enrolled, samples from 628 patients were obtained within 1 year after written informed consent was obtained and previously stocked samples from 105 patients were provided from each institute to the Japan MS/NMOSD biobank (6 patients lacked sampling date information). The collected demographic data and clinical information were as follows: gender, age at onset and registration, date and residence at clinical evaluation, EDSS scores^29^, FS scores^29^, number of relapses, relapse sites (based on clinical topography), presence of anti-AQP4 antibodies, particular MRI findings, such as a large brain lesion (> 3 cm) or a LESCL, presence of OCBs and IgG index, presence of co-existing autoimmune diseases and autoantibodies, and therapy conditions, including whether patients had been exposed to DMDs, corticosteroids or other immunosuppressant agents. PI (EDSS score/disease duration) and ARR (number of relapses/disease duration) were calculated. For MS, disease course (RRMS, SPMS or PPMS) and the MSSS^31^ were also assessed. Based on previous studies, “benign course” was defined following the most popular definitions, in which the EDSS cut-off point was ≤ 2.0 or 3.0 after disease duration of at least 10 years^43,62^. In the subanalysis, participants were stratified by their resident area into two groups (northern and southern Japan) at a latitude of 37° North, as previously described^1^. Ethical committees of National Institutes of Biomedical Innovation, Health and Nutrition, Kyushu University, National Hospital Organization Hokkaido Medical Center, Saitama Medical Center, National Hospital Organization Utano National Hospital, Ehime University Graduate School of Medicine, Yamaguchi University Graduate School of Medicine, Tokyo Medical and Dental University, Kanazawa Medical University, Kindai University, Iwate Medical University, Niigata University, Tokyo Women's Medical University Yachiyo Medical Center, Sapporo Medical University School of Medicine, Kitasato University School of Medicine, Osaka University Graduate School of Medicine, Hiroshima University, National Hospital Organization Omuta National Hospital, The University of Tokyo, Tokyo Women’s Medical University, National Hospital Organization Nagasaki Kawatana Medical Center and University of Occupational and Environmental Health School of Medicine reviewed and approved this study. This study was conducted according to the World Medical Association Declaration of Helsinki. All participants provided written informed consent between September 2013 and June 2017.

### Genotyping

The participants’ genotypes of *HLA-DRB1* and *-DPB1* alleles were determined by the sequence-specific oligonucleotide probe method (Luminex method) using DNA samples at the National Institutes of Biomedical Innovation, Health and Nutrition and the HLA Laboratory (Kyoto, Japan), as described previously^16,63^. The carrier frequencies of Japanese healthy controls from a Japan Multiple Sclerosis Genetics Consortium study were used^13^.

### Statistical analysis

To compare the demographic characteristics and clinical information between two groups, Fisher’s exact test was used for categorical data, whereas the Wilcoxon rank sum test was used for continuous data. To identify the susceptibility and protective alleles of *HLA-DRB1* and *-DPB1*, the phenotypic frequencies of these alleles were compared using Fisher’s exact test. Uncorrected *p* values (*p*^*uncorr*^) were multiplied by the number of comparisons (Bonferroni-Dunn’s correction) to calculate *p*^*corr*^ values. To calculate the risk of optic neuritis and FS scores (≥ 1) of visual function for MS patients with HLA-DRB1*04:05, logistic regression analyses were used with adjustment for disease duration. When comparing the proportions of disease course between groups, a likelihood ratio chi-square test was employed as it includes more than two categories. To identify independent factors that correlate with disease disability, EDSS scores were rank-normalized and multivariable linear regression analyses were performed. All analyses were carried out using JMP version 14.1.0 (SAS Institute, Cary, NC, USA). Statistical significance was set at *p* < 0.05.

## Supplementary Information

Supplementary Information.

## Data Availability

The datasets generated and/or analysed during the present study are available from the corresponding author based on the guidelines of the ethics committees upon reasonable request from any qualified investigator.

## Abbreviations

ANA: Anti-nuclear antibody
AQP4: Aquaporin 4
ARR: Annualized relapse rate
CI: Confidence interval
CNS: Central nervous system
CSF: Cerebrospinal fluid
DMD: Disease-modifying drug
EDSS: Expanded disability status scale
FS: Functional system
HLA: Human leukocyte antigen
LESCL: Longitudinally extensive spinal cord lesion
MOG: Myelin oligodendrocyte glycoprotein
MS: Multiple sclerosis
MSSS: Multiple sclerosis severity score
NMO: Neuromyelitis optica
NMOSD: Neuromyelitis optica spectrum disorders
OCBs: Oligoclonal IgG bands
OR: Odds ratio
PI: Progression Index
PPMS: Primary progressive multiple sclerosis
RRMS: Relapsing–remitting multiple sclerosis
SPMS: Secondary progressive multiple sclerosis
