# Supplementary material for: HLA genotype-clinical phenotype correlations in multiple sclerosis and neuromyelitis optica spectrum disorders based on Japan MS/NMOSD Biobank data
Source: Sci Rep. 2021 Jan 12;11:607. doi: 10.1038/s41598-020-79833-7 (PMC7804194; doi:10.1038/s41598-020-79833-7)
Supplement: Supplementary file 1 — Supplementary Information. [file 41598_2020_79833_MOESM1_ESM.pdf]

Supplementary Information

HLA genotype-clinical phenotype correlations in multiple sclerosis and neuromyelitis optica spectrum disorders based on Japan MS/NMOSD Biobank data

Mitsuru Watanabe, Yuri Nakamura, Shinya Sato, Masaaki Niino, Hikoaki Fukaura, Masami Tanaka, Hirofumi Ochi, Takashi Kanda, Yukio Takeshita, Takanori Yokota, Yoichiro Nishida, Makoto Matsui, Shigemi Nagayama, Susumu Kusunoki, Katsuichi Miyamoto, Masanori Mizuno, Izumi Kawachi, Etsuji Saji, Takashi Ohashi, Shun Shimohama, Shin Hisahara, Kazutoshi Nishiyama, Takahiro Iizuka, Yuji Nakatsuji, Tatsusada Okuno, Kazuhide Ochi, Akio Suzumura, Ken Yamamoto, Yuji Kawano, Shoji Tsuji, Makoto Hirata, Ryuichi Sakate, Tomonori Kimura, Yuko Shimizu, Akiko Nagaishi, Kazumasa Okada, Fumie Hayashi, Ayako Sakoda, Katsuhisa Masaki, Koji Shinoda, Noriko Isobe, Takuya Matsushita & Jun-ichi Kira*

Supplementary Table S1. Comparison of the clinical characteristics of MS patients between northern and southern Japan.

	**MS**		
	**Northern (n = 125)**	**Southern (n = 403)**	***p***
Female	88/125 (70.4)	280/403 (69.5)	0.912
Age at registration, y	40 [35-48]	41 [34-49]	0.481
Course (RR/SP/PP)	102/23/0 (81.6/18.4/0)	336/47/19 (83.6/11.7/4.7)	**0.001**
Age at onset, y	30 [23-38]	30 [23-39]	0.812
Duration of disease, y	7.5 [4.1-14.2]	9.3 [4.8-15.1]	0.297
Number of relapses	1 [1-2]	2 [1-4]	**<.001**
Annualized relapse rate	0.21 [0.08-0.42]	0.29 [0.14-0.60]	**0.001**
Lesion sites			
Optic nerve (optic neuritis)	45/123 (36.6)	146/396 (36.9)	1
Cerebrum	96/125 (76.8)	249/396 (62.9)	**0.005**
Cerebellum	26/121 (21.5)	83/396 (21.0)	0.899
Brainstem	63/122 (51.6)	191/395 (48.4)	0.536
Spinal cord	93/124 (75.0)	303/396 (76.5)	0.719
Patients with family history of MS or NMOSD	0/125 (0)	9/392 (2.3)	0.122
CSF IgG abnormality	76/102 (74.5)	233/338 (68.9)	0.324
Presence of OCBs	60/94 (63.8)	202/317 (63.7)	1
High IgG index (> 0.658)	52/77 (67.5)	160/297 (53.9)	**0.039**
Anti-AQP4 Ab positivity	1/109 (0.9)	3/345 (0.9)	1
EDSS score	2.0 [1.0-4.5]	2.0 [1.0-3.5]	0.345
PI	0.25 [0.10-0.52]	0.21 [0.06-0.48]	0.117
MSSS	3.18 [0.94-6.25]	2.44 [0.67-5.68]	0.133
FS score*			
Visual functions	0 [0-0]	0 [0-1]	0.175
Brainstem functions	0 [0-2]	0 [0-2]	0.074
Pyramidal functions	0 [0-3]	1 [0-3]	0.156
Cerebellar functions	0 [0-2]	0 [0-2]	**0.050**
Sensory functions	0 [0-2]	0 [0-3]	0.144
Bowel and bladder functions	0 [0-2]	0 [0-1]	0.881
Cerebral functions	0 [0-1]	0 [0-1]	0.514

Episodes of			
bilateral optic neuritis	7/123 (5.7)	20/393 (5.1)	0.817
transverse myelitis	3/124 (2.4)	38/389 (9.8)	**0.007**
MRI			
Huge brain lesion > 3cm	2/125 (1.6)	6/397 (1.5)	1
LESCL	1/125 (0.8)	9/398 (2.3)	0.464
Benign course			
EDSS ≤ 2 and disease duration ≥ 10y	19/52 (36.5)	82/182 (45.1)	0.341
EDSS ≤ 3 and disease duration ≥ 10y	21/52 (40.4)	106/182 (58.2)	**0.027**
AID/autoantibodies	9/125 (7.2)	68/369 (18.4)	**0.003**
AID	8/125 (6.4)	23/366 (6.3)	1
Sjögren syndrome	0/125 (0)	7/366 (1.9)	0.199
Thyroid diseases (Basedow, Hashimoto)	5/125 (4.0)	10/366 (2.7)	0.547
Autoantibodies	6/123 (4.9)	60/361 (16.6)	**0.001**
ANA	0/123 (0)	26/361 (7.2)	**0.001**
Anti-SS-A / SS-B Ab	0/123 (0)	14/361 (3.9)	**0.026**
Anti-Tg / TPO / TSHR Ab	5/123 (4.1)	21/361 (5.8)	0.643
PSL/IS	16/125 (12.8)	53/401 (13.2)	1
PSL	16/124 (12.9)	41/385 (10.6)	0.513
IS	3/124 (2.4)	21/392 (5.4)	0.225
DMD	115/125 (92.0)	344/403 (85.4)	0.067
IFN-β	90/125 (72.0)	271/403 (67.2)	0.379
fingolimod	55/125 (44.0)	162/400 (40.5)	0.533

Values are median [interquartile ranges] or count (%). Bold values indicate significant differences.

*In the section of functional system scores, values are median [10-90th percentile].

Ab, antibody; AID, autoimmune disease; ANA, anti-nuclear antibody; AQP4, aquaporin 4; CSF, cerebrospinal fluid; EDSS, expanded disability status scale; FS, functional system; IFN, interferon; IS, immunosuppressants; LESCL, longitudinally extensive spinal cord lesion; MS, multiple sclerosis; MSSS, multiple sclerosis severity score; NA, not applicable; NMOSD, neuromyelitis optica spectrum disorders; OCBs, oligoclonal IgG bands; PI, progression index; PP, primary progressive; PSL, prednisolone; RR, relapsing-remitting; SP, secondary progressive; Tg, thyroglobulin; TPO, thyroid peroxidase; TSHR, thyroid stimulating hormone receptor; y, years.

Supplementary Table S2. Comparison of the clinical characteristics between MS patients with and without *HLA-DRB1*04:05* in northern Japan.

	HLA-DRB1*04:05		
	**Positive (n = 51)**	**Negative (n = 74)**	***p***
Female	36/51 (70.6)	52/74 (70.3)	1
Age at registration, y	40 [33-50]	40 [35-47]	0.777
Course (RR/SP/PP)	39/12/0 (76.5/23.5/0)	63/11/0 (85.1/14.9/0)	0.246
Age at onset, y	30 [23-38]	30 [23-39]	0.895
Duration of disease, y	8.7 [5.6-15.1]	6.6 [3.8-13.7]	0.176
Number of relapses	1 [1-3]	1 [1-2]	0.462
Annualized relapse rate	0.17 [0.08-0.41]	0.24 [0.07-0.43]	0.564
Lesion sites			
Optic nerve (optic neuritis)	21/50 (42.0)	24/73 (32.9)	0.343
Cerebrum	39/51 (76.5)	57/74 (77.0)	1
Cerebellum	11/49 (22.4)	15/72 (20.8)	0.826
Brainstem	24/49 (49.0)	39/73 (53.4)	0.713
Spinal cord	42/51 (82.4)	51/73 (69.9)	0.142
Patients with family history of MS or NMOSD	0/51 (0)	0/74 (0)	NA
CSF IgG abnormality	25/42 (59.5)	51/60 (85.0)	**0.005**
Presence of OCBs	16/39 (41.0)	44/55 (80.0)	**<.001**
High IgG index (> 0.658)	16/33 (48.5)	36/44 (81.8)	**0.003**
Anti-AQP4 Ab positivity	0/44 (0)	1/65 (1.5)	1
EDSS score	3.0 [1.0-5.0]	2.0 [1.0-4.0]	0.183
PI	0.24 [0.12-0.53]	0.27 [0.08-0.50]	0.910
MSSS	4.83 [1.04-6.82]	2.85 [0.73-5.97]	0.246
FS score*			
Visual functions	0 [0-1]	0 [0-0]	0.102
Brainstem functions	0 [0-2]	0 [0-1]	0.296
Pyramidal functions	1 [0-3]	0 [0-3]	**0.029**
Cerebellar functions	0 [0-2]	0 [0-2]	0.519
Sensory functions	0 [0-2]	0 [0-2]	0.614
Bowel and bladder functions	0 [0-2]	0 [0-2]	0.537
Cerebral functions	0 [0-1]	0 [0-0]	0.331

Episodes of			
bilateral optic neuritis	4/50 (8.0)	3/73 (4.1)	0.441
transverse myelitis	0/50 (0)	3/74 (4.1)	0.272
MRI			
Huge brain lesion > 3cm	1/51 (2.0)	1/74 (1.4)	1
LESCL	0/51 (0)	1/74 (1.4)	1
Benign course			
EDSS ≤ 2 and disease duration ≥ 10y	8/25 (32.0)	11/27 (40.7)	0.574
EDSS ≤ 3 and disease duration ≥ 10y	9/25 (36.0)	12/27 (44.4)	0.582
AID/autoantibodies	4/51 (7.8)	5/74 (6.8)	1
AID	3/51 (5.9)	5/74 (6.8)	1
Sjögren syndrome	0/51 (0)	0/74 (0)	
Thyroid diseases (Basedow, Hashimoto)	3/51 (5.9)	2/74 (2.7)	0.397
Autoantibodies	4/51 (7.8)	2/72 (2.8)	0.231
ANA	0/51 (0)	0/72 (0)	NA
Anti-SS-A / SS-B Ab	0/51 (0)	0/72 (0)	NA
Anti-Tg / TPO / TSHR Ab	4/51 (7.8)	1/72 (1.4)	0.159
PSL/IS	5/51 (9.8)	11/74 (14.9)	0.587
PSL	5/50 (10.0)	11/74 (14.9)	0.587
IS	0/50 (0)	3/74 (4.1)	0.272
DMD	49/51 (96.1)	66/74 (89.2)	0.198
IFN-β	40/51 (78.4)	50/74 (67.6)	0.226
fingolimod	30/51 (58.8)	25/74 (33.8)	**0.006**

Values are median [interquartile ranges] or count (%). Bold values indicate significant differences.

*In the section of functional system scores, values are median [10-90th percentile].

Ab, antibody; AID, autoimmune disease; ANA, anti-nuclear antibody; AQP4, aquaporin 4; CSF, cerebrospinal fluid; EDSS, expanded disability status scale; FS, functional system; IFN, interferon; IS, immunosuppressants; LESCL, longitudinally extensive spinal cord lesion; MS, multiple sclerosis; MSSS, multiple sclerosis severity score; NA, not applicable; NMOSD, neuromyelitis optica spectrum disorders; OCBs, oligoclonal IgG bands; PI, progression index; PP, primary progressive; PSL, prednisolone; RR, relapsing-remitting; SP, secondary progressive; Tg, thyroglobulin; TPO, thyroid peroxidase; TSHR, thyroid stimulating hormone receptor; y, years.

Supplementary Table S3. Comparison of the clinical characteristics between MS patients with and without *HLA-DRB1*04:05* in southern Japan.

	HLA-DRB1*04:05		
	**Positive (n = 163)**	**Negative (n = 240)**	***p***
Female	110/163 (67.5)	170/240 (70.8)	0.509
Age at registration, y	41 [32-52]	42 [35-48]	0.740
Course (RR/SP/PP)	135/18/10 (82.8/11.0/6.1)	201/29/9 (84.1/12.1/3.8)	0.537
Age at onset, y	28 [22-38]	32 [25-39]	**0.009**
Duration of disease, y	10.3 [5.8-18]	8.7 [4.4-14.1]	**0.012**
Number of relapses	3 [1-5]	2 [1-4]	0.167
Annualized relapse rate	0.29 [0.13-0.57]	0.29 [0.15-0.64]	0.565
Lesion sites			
Optic nerve (optic neuritis)	68/162 (42.0)	78/234 (33.3)	0.090
Cerebrum	103/161 (64.0)	146/235 (62.1)	0.751
Cerebellum	37/161 (23.0)	46/235 (19.6)	0.452
Brainstem	87/161 (54.0)	104/234 (44.4)	0.066
Spinal cord	121/160 (75.6)	182/236 (77.1)	0.809
Patients with family history of MS or NMOSD	5/160 (3.1)	4/232 (1.7)	0.496
CSF IgG abnormality	78/137 (56.9)	155/201 (77.1)	**<.001**
Presence of OCBs	64/127 (50.4)	138/190 (72.6)	**<.001**
High IgG index (> 0.658)	48/118 (40.7)	112/179 (62.6)	**<.001**
Anti-AQP4 Ab positivity	1/138 (0.7)	2/207 (1.0)	1
EDSS score	2.0 [1.0-4.0]	2.0 [1.0-3.5]	0.823
PI	0.18 [0.05-0.39]	0.24 [0.08-0.49]	0.074
MSSS	2.13 [0.53-5.5]	2.82 [0.82-5.75]	0.137
FS score*			
Visual functions	0 [0-2]	0 [0-0]	**0.003**
Brainstem functions	0 [0-2]	0 [0-2]	0.649
Pyramidal functions	1 [0-3]	1 [0-3]	0.380
Cerebellar functions	0 [0-3]	0 [0-2]	0.207
Sensory functions	0 [0-3]	0 [0-3]	0.994
Bowel and bladder functions	0 [0-1]	0 [0-1]	0.938
Cerebral functions	0 [0-1]	0 [0-1]	0.786

Episodes of			
bilateral optic neuritis	11/158 (7)	9/235 (3.8)	0.241
transverse myelitis	16/156 (10.3)	22/233 (9.4)	0.862
MRI			
Huge brain lesion > 3cm	4/161 (2.5)	2/236 (0.8)	0.228
LESCL	3/163 (1.8)	6/235 (2.6)	0.743
Benign course			
EDSS ≤ 2 and disease duration ≥ 10y	41/86 (47.7)	41/96 (42.7)	0.552
EDSS ≤ 3 and disease duration ≥ 10y	49/86 (57.0)	57/96 (59.4)	0.765
AID/autoantibodies	31/149 (20.8)	37/220 (16.8)	0.341
AID	9/147 (6.1)	14/219 (6.4)	1
Sjögren syndrome	1/147 (0.7)	6/219 (2.7)	0.250
Thyroid diseases (Basedow, Hashimoto)	3/147 (2)	7/219 (3.2)	0.746
Autoantibodies	27/145 (18.6)	33/216 (15.3)	0.471
ANA	15/145 (10.3)	11/216 (5.1)	0.064
Anti-SS-A / SS-B Ab	4/145 (2.8)	10/216 (4.6)	0.419
Anti-Tg / TPO / TSHR Ab	9/145 (6.2)	12/216 (5.6)	0.821
PSL/IS	23/161 (14.3)	30/240 (12.5)	0.653
PSL	17/155 (11.0)	24/230 (10.4)	0.868
IS	11/159 (6.9)	10/233 (4.3)	0.264
DMD	136/163 (83.4)	208/240 (86.7)	0.391
IFN-β	104/163 (63.8)	167/240 (69.6)	0.236
fingolimod	60/161 (37.3)	102/239 (42.7)	0.300

Values are median [interquartile ranges] or count (%). Bold values indicate significant differences.

*In the section of functional system scores, values are median [10-90th percentile].

Ab, antibody; AID, autoimmune disease; ANA, anti-nuclear antibody; AQP4, aquaporin 4; CSF, cerebrospinal fluid; EDSS, expanded disability status scale; FS, functional system; IFN, interferon; IS, immunosuppressants; LESCL, longitudinally extensive spinal cord lesion; MS, multiple sclerosis; MSSS, multiple sclerosis severity score; NMOSD, neuromyelitis optica spectrum disorders; OCBs, oligoclonal IgG bands; PI, progression index; PP, primary progressive; PSL, prednisolone; RR, relapsing-remitting; SP, secondary progressive; Tg, thyroglobulin; TPO, thyroid peroxidase; TSHR, thyroid stimulating hormone receptor; y, years.

Supplementary Table S4. Comparison of the clinical characteristics between MS patients with and without *HLA-DRB1*04:05* in southern Japan excluding PPMS patients.

	HLA-DRB1*04:05		
	**Positive (n = 153)**	**Negative (n = 231)**	***p***
Female	108/153 (70.6)	165/231 (71.4)	0.909
Age at registration, y	41 [32-52]	41 [35-48]	0.647
Course (RR/SP)	135/18 (88.2/11.8)	201/29 (87.4/12.6)	0.874
Age at onset, y	27 [21-38]	32 [25-39]	**0.002**
Duration of disease, y	10.9 [6.0-18.5]	8.5 [4.1-14.1]	**0.003**
Number of relapses	3 [1-5]	2 [1-4]	0.083
Annualized relapse rate	0.29 [0.16-0.59]	0.30 [0.15-0.65]	0.748
Lesion sites			
Optic nerve (optic neuritis)	67/153 (43.8)	77/227 (33.9)	0.053
Cerebrum	99/152 (65.1)	141/228 (61.8)	0.587
Cerebellum	34/152 (22.4)	43/228 (18.9)	0.436
Brainstem	83/152 (54.6)	100/227 (44.1)	**0.047**
Spinal cord	115/151 (76.2)	176/229 (76.9)	0.902
Patients with family history of MS or NMOSD	5/150 (3.3)	4/223 (1.8)	0.494
CSF IgG abnormality	73/127 (57.5)	147/192 (76.6)	**0.001**
Presence of OCBs	59/118 (50.0)	130/181 (71.8)	**<.001**
High IgG index (> 0.658)	44/108 (40.7)	106/172 (61.6)	**0.001**
Anti-AQP4 Ab positivity	1/129 (0.8)	2/198 (1.0)	1
EDSS score	1.5 [1.0-3.5]	2.0 [1.0-3.0]	0.601
PI	0.16 [0.05-0.34]	0.23 [0.07-0.48]	**0.022**
MSSS	1.98 [0.51-4.46]	2.65 [0.76-5.42]	**0.050**
FS score*			
Visual functions	0 [0-2]	0 [0-0]	**0.001**
Brainstem functions	0 [0-2]	0 [0-2]	0.867
Pyramidal functions	1 [0-3]	1 [0-3]	0.181
Cerebellar functions	0 [0-3]	0 [0-2]	0.330
Sensory functions	0 [0-3]	0 [0-3]	0.841
Bowel and bladder functions	0 [0-1]	0 [0-1]	0.809
Cerebral functions	0 [0-1]	0 [0-1]	0.920

Episodes of			
bilateral optic neuritis	10/148 (6.8)	9/228 (3.9)	0.237
transverse myelitis	14/147 (9.5)	22/225 (9.8)	1
MRI			
Huge brain lesion > 3cm	3/151 (2.0)	2/228 (0.9)	0.391
LESCL	3/153 (2.0)	6/226 (2.7)	0.745
Benign course			
EDSS ≤ 2 and disease duration ≥ 10y	41/84 (48.8)	41/90 (45.6)	0.761
EDSS ≤ 3 and disease duration ≥ 10y	49/84 (58.3)	57/90 (63.3)	0.536
AID/autoantibodies	30/140 (21.4)	36/212 (17.0)	0.330
AID	9/138 (6.5)	14/211 (6.6)	1
Sjögren syndrome	1/138 (0.7)	6/211 (2.8)	0.252
Thyroid diseases (Basedow, Hashimoto)	3/138 (2.2)	7/211 (3.3)	0.746
Autoantibodies	26/136 (19.1)	32/208 (15.4)	0.380
ANA	14/136 (10.3)	11/208 (5.3)	0.092
Anti-SS-A / SS-B Ab	3/136 (2.2)	9/208 (4.3)	0.377
Anti-Tg / TPO / TSHR Ab	9/136 (6.6)	12/208 (5.8)	0.819
PSL/IS	21/151 (13.9)	29/231 (12.6)	0.757
PSL	16/145 (11)	23/221 (10.4)	0.864
IS	10/149 (6.7)	10/224 (4.5)	0.357
DMD	127/153 (83.0)	201/231 (87.0)	0.303
IFN-β	98/153 (64.1)	162/231 (70.1)	0.222
fingolimod	56/151 (37.1)	99/230 (43.0)	0.286

Values are median [interquartile ranges] or count (%). Bold values indicate significant differences.

*In the section of functional system scores, values are median [10-90th percentile].

Ab, antibody; AID, autoimmune disease; ANA, anti-nuclear antibody; AQP4, aquaporin 4; CSF, cerebrospinal fluid; EDSS, expanded disability status scale; FS, functional system; IFN, interferon; IS, immunosuppressants; LESCL, longitudinally extensive spinal cord lesion; MS, multiple sclerosis; MSSS, multiple sclerosis severity score; NMOSD, neuromyelitis optica spectrum disorders; OCBs, oligoclonal IgG bands; PI, progression index; PPMS, primary progressive multiple sclerosis; PSL, prednisolone; RR, relapsing-remitting; SP, secondary progressive; Tg, thyroglobulin; TPO, thyroid peroxidase; TSHR, thyroid stimulating hormone receptor; y, years.

Supplementary Table S5. Comparison of the clinical characteristics of MS patients with *HLA-DRB1*04:05* between northern and southern Japan excluding PPMS patients.

	**MS with *HLA-DRB1*04:05***		
	**Northern (n = 51)**	**Southern (n = 153)**	***p***
Female	36/51 (70.6)	108/153 (70.6)	1
Age at registration, y	40 [33-50]	41 [32-52]	0.937
Course (RR/SP)	39/12 (76.5/23.5)	135/18 (88.2/11.8)	0.065
Age at onset, y	30 [23-38]	27 [21-38]	0.320
Duration of disease, y	8.7 [5.6-15.1]	10.9 [6.0-18.5]	0.254
Number of relapses	1 [1-3]	3 [1-5]	**<.001**
Annualized relapse rate	0.17 [0.08-0.41]	0.29 [0.16-0.59]	**0.006**
Lesion sites			
Optic nerve (optic neuritis)	21/50 (42.0)	67/153 (43.8)	0.871
Cerebrum	39/51 (76.5)	99/152 (65.1)	0.166
Cerebellum	11/49 (22.4)	34/152 (22.4)	1
Brainstem	24/49 (49.0)	83/152 (54.6)	0.514
Spinal cord	42/51 (82.4)	115/151 (76.2)	0.438
Patients with family history of MS or NMOSD	0/51 (0)	5/150 (3.3)	0.332
CSF IgG abnormality	25/42 (59.5)	73/127 (57.5)	0.859
Presence of OCBs	16/39 (41.0)	59/118 (50.0)	0.360
High IgG index (> 0.658)	16/33 (48.5)	44/108 (40.7)	0.547
Anti-AQP4 Ab positivity	0/44 (0)	1/129 (0.8)	1
EDSS score	3.0 [1.0-5.0]	1.5 [1.0-3.5]	0.053
PI	0.24 [0.12-0.53]	0.16 [0.05-0.34]	**0.018**
MSSS	4.83 [1.04-6.82]	1.98 [0.51-4.46]	**0.008**
FS score*			
Visual functions	0 [0-1]	0 [0-2]	0.335
Brainstem functions	0 [0-2]	0 [0-2]	0.815
Pyramidal functions	1 [0-3]	1 [0-3]	0.172
Cerebellar functions	0 [0-2]	0 [0-3]	0.382
Sensory functions	0 [0-2]	0 [0-3]	0.316
Bowel and bladder functions	0 [0-2]	0 [0-1]	0.528
Cerebral functions	0 [0-1]	0 [0-1]	0.695

Episodes of			
bilateral optic neuritis	4/50 (8.0)	10/148 (6.8)	0.755
transverse myelitis	0/50 (0)	14/147 (9.5)	**0.023**
MRI			
Huge brain lesion > 3cm	1/51 (2.0)	3/151 (2.0)	1
LESCL	0/51 (0)	3/153 (2.0)	0.575
Benign course			
EDSS ≤ 2 and disease duration ≥ 10y	8/25 (32.0)	41/84 (48.8)	0.172
EDSS ≤ 3 and disease duration ≥ 10y	9/25 (36.0)	49/84 (58.3)	0.068
AID/autoantibodies	4/51 (7.8)	30/140 (21.4)	**0.033**
AID	3/51 (5.9)	9/138 (6.5)	1
Sjögren syndrome	0/51 (0)	1/138 (0.7)	1
Thyroid diseases (Basedow, Hashimoto)	3/51 (5.9)	3/138 (2.2)	0.346
Autoantibodies	4/51 (7.8)	26/136 (19.1)	0.074
ANA	0/51 (0)	14/136 (10.3)	**0.012**
Anti-SS-A / SS-B Ab	0/51 (0)	3/136 (2.2)	0.563
Anti-Tg / TPO / TSHR Ab	4/51 (7.8)	9/136 (6.6)	0.753
PSL/IS	5/51 (9.8)	21/151 (13.9)	0.629
PSL	5/50 (10.0)	16/145 (11)	1
IS	0/50 (0)	10/149 (6.7)	0.069
DMD	49/51 (96.1)	127/153 (83.0)	**0.018**
IFN-β	40/51 (78.4)	98/153 (64.1)	0.060
fingolimod	30/51 (58.8)	56/151 (37.1)	**0.009**

Values are median [interquartile ranges] or count (%). Bold values indicate significant differences.

*In the section of functional system scores, values are median [10-90th percentile].

Ab, antibody; AID, autoimmune disease; ANA, anti-nuclear antibody; AQP4, aquaporin 4; CSF, cerebrospinal fluid; EDSS, expanded disability status scale; FS, functional system; IFN, interferon; IS, immunosuppressants; LESCL, longitudinally extensive spinal cord lesion; MS, multiple sclerosis; MSSS, multiple sclerosis severity score; NMOSD, neuromyelitis optica spectrum disorders; OCBs, oligoclonal IgG bands; PI, progression index; PPMS, primary progressive multiple sclerosis; PSL, prednisolone; RR, relapsing-remitting; SP, secondary progressive; Tg, thyroglobulin; TPO, thyroid peroxidase; TSHR, thyroid stimulating hormone receptor; y, years.

Supplementary Table S6. Comparison of the clinical characteristics between MS patients with and without *HLA-DPB1*03:01*.

	HLA-DPB1*03:01		
	**Positive (n = 72)**	**Negative (n = 456)**	***p***
Female	51/72 (70.8)	317/456 (69.5)	0.891
Age at registration, y	41 [35-48]	41 [34-49]	0.579
Course (RR/SP/PP)	59/10/3 (81.9/13.9/4.2)	379/60/16 (83.3/13.2/3.5)	0.948
Age at onset, y	29 [23-37]	30 [23-39]	0.616
Duration of disease, y	10.3 [4.9-15.4]	9.0 [4.6-15]	0.676
Number of relapses	2 [1-5]	2 [1-4]	0.217
Annualized relapse rate	0.27 [0.12-0.78]	0.26 [0.13-0.53]	0.391
Lesion sites			
Optic nerve (optic neuritis)	27/70 (38.6)	164/449 (36.5)	0.790
Cerebrum	45/70 (64.3)	300/451 (66.5)	0.786
Cerebellum	18/70 (25.7)	91/447 (20.4)	0.344
Brainstem	40/69 (58.0)	214/448 (47.8)	0.122
Spinal cord	52/69 (75.4)	344/451 (76.3)	0.880
Patients with family history of MS or NMOSD	0/71 (0)	9/446 (2)	0.618
CSF IgG abnormality	40/63 (63.5)	269/377 (71.4)	0.234
Presence of OCBs	34/59 (57.6)	228/352 (64.8)	0.308
High IgG index (> 0.658)	27/55 (49.1)	185/319 (58.0)	0.240
Anti-AQP4 Ab positivity	0/61 (0)	4/393 (1)	1
EDSS score	2.0 [1.0-4.5]	2.0 [1.0-4.0]	0.694
PI	0.23 [0.09-0.52]	0.22 [0.07-0.48]	0.659
MSSS	2.44 [0.84-5.71]	2.65 [0.67-5.79]	0.864
FS score*			
Visual functions	0 [0-1]	0 [0-1]	0.282
Brainstem functions	0 [0-2]	0 [0-2]	**0.035**
Pyramidal functions	1 [0-3]	1 [0-3]	0.615
Cerebellar functions	0 [0-3]	0 [0-2]	**0.004**
Sensory functions	0 [0-2]	0 [0-3]	0.356
Bowel and bladder functions	0 [0-1]	0 [0-1]	0.597
Cerebral functions	0 [0-2]	0 [0-1]	0.098

Episodes of			
bilateral optic neuritis	5/69 (7.2)	22/447 (4.9)	0.388
transverse myelitis	5/68 (7.4)	36/445 (8.1)	1
MRI			
Huge brain lesion > 3cm	1/72 (1.4)	7/450 (1.6)	1
LESCL	1/71 (1.4)	9/452 (2.0)	1
Benign course			
EDSS ≤ 2 and disease duration ≥ 10y	14/37 (37.8)	87/197 (44.2)	0.588
EDSS ≤ 3 and disease duration ≥ 10y	18/37 (48.6)	109/197 (55.3)	0.477
AID/autoantibodies	12/66 (18.2)	65/428 (15.2)	0.584
AID	3/65 (4.6)	28/426 (6.6)	0.784
Sjögren syndrome	0/65 (0)	7/426 (1.6)	0.602
Thyroid diseases (Basedow, Hashimoto)	2/65 (3.1)	13/426 (3.1)	1
Autoantibodies	11/65 (16.9)	55/419 (13.1)	0.437
ANA	6/65 (9.2)	20/419 (4.8)	0.142
Anti-SS-A / SS-B Ab	1/65 (1.5)	13/419 (3.1)	0.705
Anti-Tg / TPO / TSHR Ab	4/65 (6.2)	22/419 (5.3)	0.767
PSL/IS	9/71 (12.7)	60/455 (13.2)	1
PSL	8/70 (11.4)	49/439 (11.2)	1
IS	4/70 (5.7)	20/446 (4.5)	0.552
DMD	63/72 (87.5)	396/456 (86.8)	1
IFN-β	51/72 (70.8)	310/456 (68.0)	0.684
fingolimod	23/71 (32.4)	194/454 (42.7)	0.120

Values are median [interquartile ranges] or count (%). Bold values indicate significant differences.

*In the section of functional system scores, values are median [10-90th percentile].

Ab, antibody; AID, autoimmune disease; ANA, anti-nuclear antibody; AQP4, aquaporin 4; CSF, cerebrospinal fluid; EDSS, expanded disability status scale; FS, functional system; IFN, interferon; IS, immunosuppressants; LESCL, longitudinally extensive spinal cord lesion; MS, multiple sclerosis; MSSS, multiple sclerosis severity score; NMOSD, neuromyelitis optica spectrum disorders; OCBs, oligoclonal IgG bands; PI, progression index; PP, primary progressive; PSL, prednisolone; RR, relapsing-remitting; SP, secondary progressive; Tg, thyroglobulin; TPO, thyroid peroxidase; TSHR, thyroid stimulating hormone receptor; y, years.

Supplementary Table S7. Comparison of the clinical characteristics between MS patients with and without *HLA-DRB1*01:01*.

	HLA-DRB1*01:01		
	**Positive (n = 29)**	**Negative (n = 499)**	***p***
Female	13/29 (44.8)	355/499 (71.1)	**0.006**
Age at registration, y	40 [35-50]	41 [34-49]	0.705
Course (RR/SP/PP)	24/3/2 (82.8/10.3/6.9)	414/67/17 (83.1/13.5/3.4)	0.621
Age at onset, y	35 [23-43]	30 [23-38]	0.257
Duration of disease, y	8.6 [3.7-14.9]	9.1 [4.6-15.1]	0.701
Number of relapses	1 [1-6]	2 [1-4]	0.580
Annualized relapse rate	0.33 [0.06-0.56]	0.26 [0.13-0.55]	0.627
Lesion sites			
Optic nerve (optic neuritis)	8/28 (28.6)	183/491 (37.3)	0.424
Cerebrum	17/28 (60.7)	328/493 (66.5)	0.542
Cerebellum	5/28 (17.9)	104/489 (21.3)	0.814
Brainstem	15/28 (53.6)	239/489 (48.9)	0.700
Spinal cord	16/28 (57.1)	380/492 (77.2)	**0.022**
Patients with family history of MS or NMOSD	0/29 (0)	9/488 (1.8)	1
CSF IgG abnormality	13/23 (56.5)	296/417 (71.0)	0.161
Presence of OCBs	13/22 (59.1)	249/389 (64.0)	0.653
High IgG index (> 0.658)	8/19 (42.1)	204/355 (57.5)	0.236
Anti-AQP4 Ab positivity	0/24 (0)	4/430 (0.9)	1
EDSS score	2.0 [1.0-3.5]	2.0 [1.0-4.0]	0.642
PI	0.21 [0.08-0.39]	0.22 [0.07-0.49]	0.718
MSSS	2.70 [0.70-3.67]	2.65 [0.67-5.87]	0.561
FS score*			
Visual functions	0 [0-0]	0 [0-1]	0.423
Brainstem functions	0 [0-1]	0 [0-2]	0.153
Pyramidal functions	1 [0-3]	1 [0-3]	0.431
Cerebellar functions	0 [0-2]	0 [0-2]	0.601
Sensory functions	0 [0-2]	0 [0-2]	**0.048**
Bowel and bladder functions	0 [0-2]	0 [0-1]	0.415
Cerebral functions	0 [0-0]	0 [0-1]	0.174

Episodes of			
bilateral optic neuritis	3/28 (10.7)	24/488 (4.9)	0.175
transverse myelitis	0/29 (0)	41/484 (8.5)	0.155
MRI			
Huge brain lesion > 3cm	0/29 (0)	8/493 (1.6)	1
LESCL	0/29 (0)	10/494 (2.0)	1
Benign course			
EDSS ≤ 2 and disease duration ≥ 10y	3/11 (27.3)	98/223 (43.9)	0.358
EDSS ≤ 3 and disease duration ≥ 10y	5/11 (45.5)	122/223 (54.7)	0.555
AID/autoantibodies	6/26 (23.1)	71/468 (15.2)	0.270
AID	4/26 (15.4)	27/465 (5.8)	0.073
Sjögren syndrome	0/26 (0)	7/465 (1.5)	1
Thyroid diseases (Basedow, Hashimoto)	1/26 (3.8)	14/465 (3.0)	0.563
Autoantibodies	3/25 (12.0)	63/459 (13.7)	1
ANA	1/25 (4.0)	25/459 (5.4)	1
Anti-SS-A / SS-B Ab	0/25 (0)	14/459 (3.1)	1
Anti-Tg / TPO / TSHR Ab	1/25 (4.0)	25/459 (5.4)	1
PSL/IS	4/29 (13.8)	65/497 (13.1)	0.783
PSL	2/28 (7.1)	55/481 (11.4)	0.757
IS	3/28 (10.7)	21/488 (4.3)	0.135
DMD	22/29 (75.9)	437/499 (87.6)	0.085
IFN-β	17/29 (58.6)	344/499 (68.9)	0.304
fingolimod	11/28 (39.3)	206/497 (41.4)	1

Values are median [interquartile ranges] or count (%). Bold values indicate significant differences.

*In the section of functional system scores, values are median [10-90th percentile].

Ab, antibody; AID, autoimmune disease; ANA, anti-nuclear antibody; AQP4, aquaporin 4; CSF, cerebrospinal fluid; EDSS, expanded disability status scale; FS, functional system; IFN, interferon; IS, immunosuppressants; LESCL, longitudinally extensive spinal cord lesion; MS, multiple sclerosis; MSSS, multiple sclerosis severity score; NMOSD, neuromyelitis optica spectrum disorders; OCBs, oligoclonal IgG bands; PI, progression index; PP, primary progressive; PSL, prednisolone; RR, relapsing-remitting; SP, secondary progressive; Tg, thyroglobulin; TPO, thyroid peroxidase; TSHR, thyroid stimulating hormone receptor; y, years.

Supplementary Table S8. Comparison of the clinical characteristics between MS patients with and without *HLA-DRB1*09:01*.

	HLA-DRB1*09:01		
	**Positive (n = 76)**	**Negative (n = 452)**	***p***
Female	56/76 (73.7)	312/452 (69.0)	0.500
Age at registration, y	43 [33-50]	41 [34-49]	0.664
Course (RR/SP/PP)	67/5/3 (89.3/6.7/4.0)	371/65/16 (82.1/14.4/3.5)	0.145
Age at onset, y	31 [23-39]	30 [23-38]	0.437
Duration of disease, y	9.0 [4.4-15.0]	9.1 [4.7-15.1]	0.983
Number of relapses	2 [1-3]	2 [1-4]	0.062
Annualized relapse rate	0.23 [0.11-0.43]	0.28 [0.14-0.58]	**0.047**
Lesion sites			
Optic nerve (optic neuritis)	26/74 (35.1)	165/445 (37.1)	0.796
Cerebrum	43/74 (58.1)	302/447 (67.6)	0.114
Cerebellum	11/73 (15.1)	98/444 (22.1)	0.216
Brainstem	24/72 (33.3)	230/445 (51.7)	**0.005**
Spinal cord	52/74 (70.3)	344/446 (77.1)	0.238
Patients with family history of MS or NMOSD	1/74 (1.4)	8/443 (1.8)	1
CSF IgG abnormality	43/63 (68.3)	266/377 (70.6)	0.766
Presence of OCBs	34/55 (61.8)	228/356 (64)	0.765
High IgG index (> 0.658)	32/51 (62.7)	180/323 (55.7)	0.366
Anti-AQP4 Ab positivity	0/65 (0)	4/389 (1.0)	1
EDSS score	1.8 [1.0-3.0]	2.0 [1.0-4.0]	0.434
PI	0.21 [0.07-0.49]	0.22 [0.07-0.49]	0.913
MSSS	2.19 [0.66-5.38]	2.72 [0.67-5.87]	0.412
FS score*			
Visual functions	0 [0-0]	0 [0-1]	0.414
Brainstem functions	0 [0-1]	0 [0-2]	0.273
Pyramidal functions	1 [0-3]	1 [0-3]	0.062
Cerebellar functions	0 [0-2]	0 [0-2]	0.131
Sensory functions	1 [0-2]	0 [0-3]	0.355
Bowel and bladder functions	0 [0-1]	0 [0-2]	**0.015**
Cerebral functions	0 [0-0]	0 [0-1]	0.242

Episodes of			
bilateral optic neuritis	3/74 (4.1)	24/442 (5.4)	0.783
transverse myelitis	6/73 (8.2)	35/440 (8.0)	1
MRI			
Huge brain lesion > 3cm	3/76 (3.9)	5/446 (1.1)	0.096
LESCL	2/75 (2.7)	8/448 (1.8)	0.642
Benign course			
EDSS ≤ 2 and disease duration ≥ 10y	16/34 (47.1)	85/200 (42.5)	0.709
EDSS ≤ 3 and disease duration ≥ 10y	23/34 (67.6)	104/200 (52.0)	0.098
AID/autoantibodies	8/69 (11.6)	69/425 (16.2)	0.375
AID	3/68 (4.4)	28/423 (6.6)	0.602
Sjögren syndrome	1/68 (1.5)	6/423 (1.4)	1
Thyroid diseases (Basedow, Hashimoto)	2/68 (2.9)	13/423 (3.1)	1
Autoantibodies	7/69 (10.1)	59/415 (14.2)	0.450
ANA	3/69 (4.3)	23/415 (5.5)	1
Anti-SS-A / SS-B Ab	1/69 (1.4)	13/415 (3.1)	0.704
Anti-Tg / TPO / TSHR Ab	2/69 (2.9)	24/415 (5.8)	0.561
PSL/IS	6/75 (8.0)	63/451 (14.0)	0.196
PSL	5/73 (6.8)	52/436 (11.9)	0.235
IS	3/75 (4.0)	21/441 (4.8)	1
DMD	64/76 (84.2)	395/452 (87.4)	0.462
IFN-β	46/76 (60.5)	315/452 (69.7)	0.142
fingolimod	31/76 (40.8)	186/449 (41.4)	1

Values are median [interquartile ranges] or count (%). Bold values indicate significant differences.

*In the section of functional system scores, values are median [10-90th percentile].

Ab, antibody; AID, autoimmune disease; ANA, anti-nuclear antibody; AQP4, aquaporin 4; CSF, cerebrospinal fluid; EDSS, expanded disability status scale; FS, functional system; IFN, interferon; IS, immunosuppressants; LESCL, longitudinally extensive spinal cord lesion; MS, multiple sclerosis; MSSS, multiple sclerosis severity score; NMOSD, neuromyelitis optica spectrum disorders; OCBs, oligoclonal IgG bands; PI, progression index; PP, primary progressive; PSL, prednisolone; RR, relapsing-remitting; SP, secondary progressive; Tg, thyroglobulin; TPO, thyroid peroxidase; TSHR, thyroid stimulating hormone receptor; y, years.

Supplementary Table S9. Comparison of the clinical characteristics between MS patients with and without *HLA-DRB1*13:02*.

	HLA-DRB1*13:02		
	**Positive (n = 15)**	**Negative (n = 513)**	***p***
Female	13/15 (86.7)	355/513 (69.2)	0.252
Age at registration, y	49 [38-54]	41 [34-49]	0.041
Course (RR/SP/PP)	425/68/19 (83.0/13.3/3.7)	13/2/0 (86.7/13.3/0)	0.571
Age at onset, y	33 [28-42]	30 [23-38]	0.153
Duration of disease, y	9.6 [6.4-15.7]	9.1 [4.6-15.1]	0.401
Number of relapses	2 [1-3]	2 [1-4]	0.791
Annualized relapse rate	0.19 [0.09-0.42]	0.27 [0.13-0.56]	0.138
Lesion sites			
Optic nerve (optic neuritis)	5/15 (33.3)	186/504 (36.9)	1
Cerebrum	9/14 (64.3)	336/507 (66.3)	1
Cerebellum	2/14 (14.3)	107/503 (21.3)	0.744
Brainstem	3/14 (21.4)	251/503 (49.9)	0.055
Spinal cord	8/15 (53.3)	388/505 (76.8)	0.058
Patients with family history of MS or NMOSD	0/13 (0)	9/504 (1.8)	1
CSF IgG abnormality	4/11 (36.4)	305/429 (71.1)	**0.019**
Presence of OCBs	2/11 (18.2)	260/400 (65.0)	**0.002**
High IgG index (> 0.658)	3/8 (37.5)	209/366 (57.1)	0.300
Anti-AQP4 Ab positivity	0/12 (0)	4/442 (0.9)	1
EDSS score	1.5 [0.0-3.5]	2.0 [1.0-4.0]	0.440
PI	0.16 [0.00-0.44]	0.22 [0.07-0.49]	0.196
MSSS	1.45 [0.24-5.75]	2.70 [0.67-5.79]	0.182
FS score[*]			
Visual functions	0 [0-1]	0 [0-1]	0.550
Brainstem functions	0 [0-1]	0 [0-2]	0.137
Pyramidal functions	0 [0-4]	1 [0-3]	0.592
Cerebellar functions	0 [0-2]	0 [0-2]	0.651
Sensory functions	0 [0-2]	0 [0-3]	0.863
Bowel and bladder functions	0 [0-1]	0 [0-1]	0.329
Cerebral functions	0 [0-0]	0 [0-1]	0.163

Episodes of			
bilateral optic neuritis	0/15 (0)	27/501 (5.4)	1
transverse myelitis	0/14 (0)	41/499 (8.2)	0.616
MRI			
Huge brain lesion > 3cm	1/15 (6.7)	7/507 (1.4)	0.209
LESCL	0/15 (0)	10/508 (2.0)	1
Benign course			
EDSS ≤ 2 and disease duration ≥ 10y	3/7 (42.9)	98/227 (43.2)	1
EDSS ≤ 3 and disease duration ≥ 10y	5/7 (71.4)	122/227 (53.7)	0.458
AID/autoantibodies	4/14 (28.6)	73/480 (15.2)	0.250
AID	3/14 (21.4)	28/477 (5.9)	0.052
Sjögren syndrome	0/14 (0)	7/477 (1.5)	1
Thyroid diseases (Basedow, Hashimoto)	1/14 (7.1)	14/477 (2.9)	0.356
Autoantibodies	1/12 (8.3)	65/472 (13.8)	1
ANA	0/12 (0)	26/472 (5.5)	1
Anti-SS-A / SS-B Ab	1/12 (8.3)	13/472 (2.8)	0.300
Anti-Tg / TPO / TSHR Ab	0/12 (0)	26/472 (5.5)	1
PSL/IS	0/15 (0)	69/511 (13.5)	0.238
PSL	0/12 (0)	57/497 (11.5)	0.378
IS	0/15 (0)	24/501 (4.8)	1
DMD	13/15 (86.7)	446/513 (86.9)	1
IFN-β	9/15 (60.0)	352/513 (68.6)	0.574
fingolimod	5/15 (33.3)	212/510 (41.6)	0.603

Values are median [interquartile ranges] or count (%). Bold values indicate significant differences.
*In the section of functional system scores, values are median [10-90th percentile].
Ab, antibody; AID, autoimmune disease; ANA, anti-nuclear antibody; AQP4, aquaporin 4; CSF, cerebrospinal fluid; EDSS, expanded disability status scale; FS, functional system; IFN, interferon; IS, immunosuppressants; LESCL, longitudinally extensive spinal cord lesion; MS, multiple sclerosis; MSSS, multiple sclerosis severity score; NMOSD, neuromyelitis optica spectrum disorders; OCBs, oligoclonal IgG bands; PI, progression index; PP, primary progressive; PSL, prednisolone; RR, relapsing-remitting; SP, secondary progressive; Tg, thyroglobulin; TPO, thyroid peroxidase; TSHR, thyroid stimulating hormone receptor; y, years.

Supplementary Table S10. Comparison of the clinical characteristics between MS patients with and without *HLA-DPB1*04:01*.

	HLA-DPB1*04:01		
	**Positive (n = 18)**	**Negative (n = 510)**	***p***
Female	15/18 (83.3)	353/510 (69.2)	0.297
Age at registration, y	42 [32-51]	41 [35-49]	0.907
Course (RR/SP/PP)	17/1/0 (94.4/5.6/0)	421/69/19 (82.7/13.6/3.7)	0.264
Age at onset, y	26 [24-39]	30 [23-38]	0.611
Duration of disease, y	8.0 [6.0-16.5]	9.1 [4.6-15.1]	0.778
Number of relapses	3 [1-4]	2 [1-4]	0.461
Annualized relapse rate	0.23 [0.15-0.5]	0.27 [0.13-0.55]	0.817
Lesion sites			
Optic nerve (optic neuritis)	5/18 (27.8)	186/501 (37.1)	0.468
Cerebrum	10/18 (55.6)	335/503 (66.6)	0.323
Cerebellum	4/18 (22.2)	105/499 (21.0)	1
Brainstem	6/18 (33.3)	248/499 (49.7)	0.231
Spinal cord	14/18 (77.8)	382/502 (76.1)	1
Patients with family history of MS or NMOSD	0/17 (0)	9/500 (1.8)	1
CSF IgG abnormality	7/15 (46.7)	302/425 (71.1)	0.079
Presence of OCBs	4/15 (26.7)	258/396 (65.2)	**0.004**
High IgG index (> 0.658)	4/13 (30.8)	208/361 (57.6)	0.084
Anti-AQP4 Ab positivity	0/16 (0)	4/438 (0.9)	1
EDSS score	2.0 [1.0-3.8]	2.0 [1.0-4.0]	0.953
PI	0.23 [0.03-0.50]	0.22 [0.07-0.49]	0.809
MSSS	2.63 [0.25-6.64]	2.65 [0.67-5.75]	0.956
FS score*			
Visual functions	0 [0-0]	0 [0-1]	0.444
Brainstem functions	0 [0-2]	0 [0-2]	0.548
Pyramidal functions	1 [0-4]	1 [0-3]	0.691
Cerebellar functions	0 [0-2]	0 [0-2]	0.776
Sensory functions	0 [0-3]	0 [0-2]	0.943
Bowel and bladder functions	0 [0-0]	0 [0-1]	0.072
Cerebral functions	0 [0-0]	0 [0-1]	0.125

Episodes of			
bilateral optic neuritis	0/17 (0)	27/499 (5.4)	1
transverse myelitis	0/18 (0)	41/495 (8.3)	0.384
MRI			
Huge brain lesion > 3cm	1/18 (5.6)	7/504 (1.4)	0.246
LESCL	2/18 (11.1)	8/505 (1.6)	**0.043**
Benign course			
EDSS ≤ 2 and disease duration ≥ 10y	3/7 (42.9)	98/227 (43.2)	1
EDSS ≤ 3 and disease duration ≥ 10y	4/7 (57.1)	123/227 (54.2)	1
AID/autoantibodies	1/18 (5.6)	76/476 (16.0)	0.332
AID	0/18 (0)	31/473 (6.6)	0.619
Sjögren syndrome	0/18 (0)	7/473 (1.5)	1
Thyroid diseases (Basedow, Hashimoto)	0/18 (0)	15/473 (3.2)	1
Autoantibodies	1/18 (5.6)	65/466 (13.9)	0.490
ANA	1/18 (5.6)	25/466 (5.4)	1
Anti-SS-A / SS-B Ab	0/18 (0)	14/466 (3.0)	1
Anti-Tg / TPO / TSHR Ab	0/18 (0)	26/466 (5.6)	0.614
PSL/IS	2/18 (11.1)	67/508 (13.2)	1
PSL	2/15 (13.3)	55/494 (11.1)	0.680
IS	1/18 (5.6)	23/498 (4.6)	0.582
DMD	16/18 (88.9)	443/510 (86.9)	1
IFN-β	11/18 (61.1)	350/510 (68.6)	0.607
fingolimod	6/18 (33.3)	211/507 (41.6)	0.628

Values are median [interquartile ranges] or count (%). Bold values indicate significant differences.

*In the section of functional system scores, values are median [10-90th percentile].

Ab, antibody; AID, autoimmune disease; ANA, anti-nuclear antibody; AQP4, aquaporin 4; CSF, cerebrospinal fluid; EDSS, expanded disability status scale; FS, functional system; IFN, interferon; IS, immunosuppressants; LESCL, longitudinally extensive spinal cord lesion; MS, multiple sclerosis; MSSS, multiple sclerosis severity score; NMOSD, neuromyelitis optica spectrum disorders; OCBs, oligoclonal IgG bands; PI, progression index; PP, primary progressive; PSL, prednisolone; RR, relapsing-remitting; SP, secondary progressive; Tg, thyroglobulin; TPO, thyroid peroxidase; TSHR, thyroid stimulating hormone receptor; y, years.

Supplementary Table S11. Comparison of the clinical characteristics between NMOSD patients with and without *HLA-DRB1*08:02*.

	HLA-DRB1*08:02		
	**Positive (n = 30)**	**Negative (n = 135)**	***p***
Female	24/30 (80.0)	119/135 (88.1)	0.242
Age at registration, y	56 [46-62]	52 [42-63]	0.914
Age at onset, y	48 [39-53]	44 [33-54]	0.410
Duration of disease, y	7.2 [4.3-10.3]	6.9 [3.2-13.7]	0.881
Number of relapses	2 [1-4]	2 [1-5]	0.474
Annualized relapse rate	0.32 [0.21-0.56]	0.43 [0.21-0.8]	0.502
Lesion sites			
Optic nerve (optic neuritis)	15/30 (50.0)	83/134 (61.9)	0.303
Cerebrum	6/30 (20.0)	33/133 (24.8)	0.644
Cerebellum	2/30 (6.7)	12/133 (9.0)	1
Brainstem	8/30 (26.7)	55/133 (41.4)	0.152
Spinal cord	25/30 (83.3)	113/134 (84.3)	1
Patients with family history of MS or NMOSD	1/30 (3.3)	4/131 (3.1)	1
CSF IgG abnormality	6/26 (23.1)	49/111 (44.1)	0.074
Presence of OCBs	0/23 (0)	20/95 (21.1)	**0.012**
High IgG index (> 0.658)	6/23 (26.1)	40/99 (40.4)	0.239
Anti-AQP4 Ab positivity	30/30 (100)	125/133 (94.0)	0.353
EDSS score	3.5 [2.0-6.0]	3.5 [2.0-5.5]	0.965
PI	0.43 [0.29-0.86]	0.42 [0.23-0.94]	0.657
FS score*			
Visual functions	0 [0-4]	0 [0-5]	0.123
Brainstem functions	0 [0-1]	0 [0-1]	0.342
Pyramidal functions	2 [0-3]	2 [0-4]	0.307
Cerebellar functions	0 [0-0]	0 [0-1]	0.402
Sensory functions	2 [0-4]	1 [0-4]	0.190
Bowel and bladder functions	0 [0-4]	0 [0-2]	0.990
Cerebral functions	0 [0-0]	0 [0-0]	0.481
Episodes of			
bilateral optic neuritis	4/29 (13.8)	25/131 (19.1)	0.603
transverse myelitis	15/29 (51.7)	49/134 (36.6)	0.146

MRI			
Huge brain lesion > 3cm	2/30 (6.7)	5/135 (3.7)	0.612
LESCL	21/29 (72.4)	72/133 (54.1)	0.097
Benign course			
EDSS ≤ 2 and disease duration ≥ 10y	1/7 (14.3)	8/43 (18.6)	1
EDSS ≤ 3 and disease duration ≥ 10y	3/7 (42.9)	10/43 (23.3)	0.357
AID/autoantibodies	17/28 (60.7)	47/127 (37.0)	**0.033**
AID	7/28 (25.0)	16/123 (13.0)	0.143
Sjögren syndrome	4/28 (14.3)	12/123 (9.8)	0.500
Thyroid diseases (Basedow, Hashimoto)	2/28 (7.1)	1/123 (0.8)	0.089
Autoantibodies	16/27 (59.3)	44/121 (36.4)	**0.032**
ANA	9/27 (33.3)	20/121 (16.5)	0.061
Anti-SS-A / SS-B Ab	10/27 (37.0)	21/121 (17.4)	**0.035**
Anti-Tg / TPO / TSHR Ab	3/27 (11.1)	11/121 (9.1)	0.721
PSL/IS	28/30 (93.3)	134/135 (99.3)	0.085
PSL	28/29 (96.6)	129/134 (96.3)	1
IS	17/30 (56.7)	94/134 (70.1)	0.195
DMD	3/30 (10.0)	24/135 (17.8)	0.416
IFN-β	3/30 (10.0)	24/135 (17.8)	0.416
fingolimod	1/30 (3.3)	0/134 (0)	0.183

Values are median [interquartile ranges] or count (%). Bold values indicate significant differences.
*In the section of functional system scores, values are median [10-90th percentile].
Ab, antibody; AID, autoimmune disease; ANA, anti-nuclear antibody; AQP4, aquaporin 4; CSF, cerebrospinal fluid; EDSS, expanded disability status scale; FS, functional system; IFN, interferon; IS, immunosuppressants; LESCL, longitudinally extensive spinal cord lesion; MS = multiple sclerosis; NMOSD, neuromyelitis optica spectrum disorders; OCBs, oligoclonal IgG bands; PI, progression index; PSL, prednisolone; Tg, thyroglobulin; TPO, thyroid peroxidase; TSHR, thyroid stimulating hormone receptor; y, years.

Supplementary Table S12. Comparison of the clinical characteristics between NMOSD patients with and without *HLA-DPB1*05:01*.

	HLA-DPB1*05:01		
	**Positive (n = 135)**	**Negative (n = 30)**	***p***
Female	118/135 (87.4)	25/30 (83.3)	0.557
Age at registration, y	53 [44-63]	50 [36-65]	0.621
Age at onset, y	45 [34-53]	45 [32-59]	0.642
Duration of disease, y	7.9 [4.1-13.1]	4.3 [1.5-8.6]	**0.016**
Number of relapses	3 [1-6]	1 [0-2]	**0.001**
Annualized relapse rate	0.43 [0.23-0.75]	0.24 [0-0.77]	0.155
Lesion sites			
Optic nerve (optic neuritis)	80/135 (59.3)	18/29 (62.1)	0.837
Cerebrum	36/134 (26.9)	3/29 (10.3)	0.090
Cerebellum	14/134 (10.4)	0/29 (0)	0.077
Brainstem	53/134 (39.6)	10/29 (34.5)	0.678
Spinal cord	115/135 (85.2)	23/29 (79.3)	0.412
Patients with family history of MS or NMOSD	5/132 (3.8)	0/29 (0)	0.586
CSF IgG abnormality	47/112 (42)	8/25 (32)	0.499
Presence of OCBs	19/99 (19.2)	1/19 (5.3)	0.191
High IgG index (> 0.658)	38/98 (38.8)	8/24 (33.3)	0.815
Anti-AQP4 Ab positivity	128/134 (95.5)	27/29 (93.1)	0.633
EDSS score	3.5 [2.0-5.0]	3.5 [1.5-6.0]	0.894
PI	0.40 [0.23-0.79]	0.83 [0.27-1.85]	**0.034**
FS score*			
Visual functions	0 [0-5]	0 [0-6]	0.896
Brainstem functions	0 [0-1]	0 [0-2]	0.860
Pyramidal functions	2 [0-3]	0 [0-4]	0.130
Cerebellar functions	0 [0-1]	0 [0-0]	0.454
Sensory functions	2 [0-4]	1 [0-4]	0.751
Bowel and bladder functions	0 [0-2]	0 [0-3]	0.544
Cerebral functions	0 [0-0]	0 [0-0]	0.915
Episodes of			
bilateral optic neuritis	23/131 (17.6)	6/29 (20.7)	0.790
transverse myelitis	56/133 (42.1)	8/30 (26.7)	0.149

MRI			
Huge brain lesion > 3cm	6/135 (4.4)	1/30 (3.3)	1
LESCL	79/132 (59.8)	14/30 (46.7)	0.222
Benign course			
EDSS ≤ 2 and disease duration ≥ 10y	8/44 (18.2)	1/6 (16.7)	1
EDSS ≤ 3 and disease duration ≥ 10y	11/44 (25)	2/6 (33.3)	0.643
AID/autoantibodies	55/127 (43.3)	9/28 (32.1)	0.299
AID	23/123 (18.7)	0/28 (0)	**0.008**
Sjögren syndrome	16/123 (13.0)	0/28 (0)	**0.044**
Thyroid diseases (Basedow, Hashimoto)	3/123 (2.4)	0/28 (0)	1
Autoantibodies	51/121 (42.1)	9/27 (33.3)	0.517
ANA	24/121 (19.8)	5/27 (18.5)	1
Anti-SS-A / SS-B Ab	30/121 (24.8)	1/27 (3.7)	**0.016**
Anti-Tg / TPO / TSHR Ab	11/121 (9.1)	3/27 (11.1)	0.721
PSL/IS	133/135 (98.5)	29/30 (96.7)	0.455
PSL	128/133 (96.2)	29/30 (96.7)	1
IS	90/134 (67.2)	21/30 (70)	0.832
DMD	25/135 (18.5)	2/30 (6.7)	0.171
IFN-β	25/135 (18.5)	2/30 (6.7)	0.171
fingolimod	1/134 (0.7)	0/30 (0)	1

Values are median [interquartile ranges] or count (%). Bold values indicate significant differences.

*In the section of functional system scores, values are median [10-90th percentile].

Ab, antibody; AID, autoimmune disease; ANA, anti-nuclear antibody; AQP4, aquaporin 4; CSF, cerebrospinal fluid; EDSS, expanded disability status scale; FS, functional system; IFN, interferon; IS, immunosuppressants; LESCL, longitudinally extensive spinal cord lesion; MS = multiple sclerosis; NMOSD, neuromyelitis optica spectrum disorders; OCBs, oligoclonal IgG bands; PI, progression index; PSL, prednisolone; Tg, thyroglobulin; TPO, thyroid peroxidase; TSHR, thyroid stimulating hormone receptor; y, years.

Supplementary Table S13. Comparison of the clinical characteristics between NMOSD patients with and without *HLA-DRB1*09:01*.

	HLA-DRB1*09:01		
	**Positive (n = 15)**	**Negative (n = 150)**	***p***
Female	14/15 (93.3)	129/150 (86.0)	0.695
Age at registration, y	59 [49-65]	52 [43-62]	0.249
Age at onset, y	45 [31-56]	45 [33-53]	0.721
Duration of disease, y	7.3 [2.4-20.7]	7.0 [3.6-12.2]	0.723
Number of relapses	2 [1-13]	2 [1-5]	0.567
Annualized relapse rate	0.42 [0.14-1.18]	0.41 [0.21-0.74]	0.765
Lesion sites			
Optic nerve (optic neuritis)	9/15 (60.0)	89/149 (59.7)	1
Cerebrum	4/15 (26.7)	35/148 (23.6)	0.757
Cerebellum	0/15 (0)	14/148 (9.5)	0.367
Brainstem	8/15 (53.3)	55/148 (37.2)	0.269
Spinal cord	11/15 (73.3)	127/149 (85.2)	0.262
Patients with family history of MS or NMOSD	0/15 (0)	5/146 (3.4)	1
CSF IgG abnormality	7/13 (53.8)	48/124 (38.7)	0.375
Presence of OCBs	4/10 (40.0)	16/108 (14.8)	0.065
High IgG index (> 0.658)	5/9 (55.6)	41/113 (36.3)	0.295
Anti-AQP4 Ab positivity	13/14 (92.9)	142/149 (95.3)	0.521
EDSS score	3.5 [1.5-7.0]	3.5 [2.0-5.5]	0.914
PI	0.42 [0.24-0.55]	0.42 [0.23-0.95]	0.553
FS score*			
Visual functions	0 [0-5]	0 [0-5]	0.943
Brainstem functions	0 [0-1]	0 [0-1]	0.275
Pyramidal functions	1 [0-5]	2 [0-3]	0.917
Cerebellar functions	0 [0-1]	0 [0-1]	0.603
Sensory functions	2 [0-4]	1 [0-3]	0.323
Bowel and bladder functions	0 [0-4]	0 [0-2]	0.374
Cerebral functions	0 [0-2]	0 [0-0]	0.875
Episodes of			
bilateral optic neuritis	2/15 (13.3)	27/145 (18.6)	1
transverse myelitis	4/15 (26.7)	60/148 (40.5)	0.408

MRI			
Huge brain lesion > 3cm	2/15 (13.3)	5/150 (3.3)	0.124
LESCL	10/15 (66.7)	83/147 (56.5)	0.586
Benign course			
EDSS ≤ 2 and disease duration ≥ 10y	1/5 (20.0)	8/45 (17.8)	1
EDSS ≤ 3 and disease duration ≥ 10y	1/5 (20.0)	12/45 (26.7)	1
AID/autoantibodies	5/13 (38.5)	59/142 (41.5)	1
AID	5/13 (38.5)	18/138 (13.0)	**0.030**
Sjögren syndrome	4/13 (30.8)	12/138 (8.7)	**0.034**
Thyroid diseases (Basedow, Hashimoto)	0/13 (0)	3/138 (2.2)	1
Autoantibodies	3/12 (25.0)	57/136 (41.9)	0.361
ANA	2/12 (16.7)	27/136 (19.9)	1
Anti-SS-A / SS-B Ab	3/12 (25.0)	28/136 (20.6)	0.716
Anti-Tg / TPO / TSHR Ab	0/12 (0)	14/136 (10.3)	0.605
PSL/IS	15/15 (100)	147/150 (98.0)	1
PSL	15/15 (100)	142/148 (95.9)	1
IS	11/14 (78.6)	100/150 (66.7)	0.552
DMD	4/15 (26.7)	23/150 (15.3)	0.274
IFN-β	4/15 (26.7)	23/150 (15.3)	0.274
fingolimod	0/15 (0)	1/149 (0.7)	1

Values are median [interquartile ranges] or count (%). Bold values indicate significant differences.
*In the section of functional system scores, values are median [10-90th percentile].
Ab, antibody; AID, autoimmune disease; ANA, anti-nuclear antibody; AQP4, aquaporin 4; CSF, cerebrospinal fluid; EDSS, expanded disability status scale; FS, functional system; IFN, interferon; IS, immunosuppressants; LESCL, longitudinally extensive spinal cord lesion; MS = multiple sclerosis; NMOSD, neuromyelitis optica spectrum disorders; OCBs, oligoclonal IgG bands; PI, progression index; PSL, prednisolone; Tg, thyroglobulin; TPO, thyroid peroxidase; TSHR, thyroid stimulating hormone receptor; y, years.

Supplementary Table S14. Clinical and genetic factors associated with EDSS scores in NMOSD.

EDSS score (ordinal scale)	Univariable		Multivariable	
	**Std β**	***p***	**Std β**	***p***
Sex [female/male]	0.063	0.418	0.006	0.944
Age at onset (y)	0.074	0.346	0.255	**0.004**
Disease Duration (y)	0.342	**<.001**	0.260	**0.008**
Number of relapses	0.380	**<.001**	0.284	**0.004**
Episode(s) of bilateral optic neuritis [+/−]	0.143	0.071	0.084	0.281
LESCL [+/−]	0.189	**0.016**	0.052	0.512
AID/autoantibody [+/−]	−0.051	0.529	−0.027	0.724
PSL/IS [treated/non-treated]	−0.060	0.443	−0.065	0.407
*HLA-DPB1*05:01* [+/−]	−0.011	0.893	−0.102	0.187

Significant correlations are in bold.

AID, autoimmune disease; EDSS, expanded disability status scale; IS, immunosuppressants; LESCL, longitudinally extensive spinal cord lesion; NMOSD, neuromyelitis optica spectrum disorders; PSL, prednisolone; Std, standardized; y, years.
